# Production of the Fragrance Geraniol in Peroxisomes of a Product-Tolerant Baker’s Yeast

**DOI:** 10.3389/fbioe.2020.582052

**Published:** 2020-09-23

**Authors:** Jennifer Gerke, Holm Frauendorf, Dominik Schneider, Maxim Wintergoller, Thomas Hofmeister, Anja Poehlein, Ziga Zebec, Eriko Takano, Nigel S. Scrutton, Gerhard H. Braus

**Affiliations:** ^1^Department of Molecular Microbiology and Genetics, Institute of Microbiology and Genetics, Georg-August-Universität Göttingen, Göttingen, Germany; ^2^Institute of Organic and Biomolecular Chemistry, Georg-August-Universität Göttingen, Göttingen, Germany; ^3^Department of Genomic and Applied Microbiology, Göttingen Genomics Laboratory, Institute of Microbiology and Genetics, Georg-August-Universität Göttingen, Göttingen, Germany; ^4^Thermo Fisher Scientific GENEART GmbH, Regensburg, Germany; ^5^Molecular Enzymology, Manchester Institute of Biotechnology, University of Manchester, Manchester, United Kingdom; ^6^Synthetic Biology Research Centre, SYNBIOCHEM, Manchester Institute of Biotechnology, University of Manchester, Manchester, United Kingdom

**Keywords:** geraniol, peroxisomes, *Saccharomyces cerevisiae*, compartmentalization, tolerance, monoterpenoids

## Abstract

Monoterpenoids, such as the plant metabolite geraniol, are of high industrial relevance since they are important fragrance materials for perfumes, cosmetics, and household products. Chemical synthesis or extraction from plant material for industry purposes are complex, environmentally harmful or expensive and depend on seasonal variations. Heterologous microbial production offers a cost-efficient and sustainable alternative but suffers from low metabolic flux of the precursors and toxicity of the monoterpenoid to the cells. In this study, we evaluated two approaches to counteract both issues by compartmentalizing the biosynthetic enzymes for geraniol to the peroxisomes of *Saccharomyces cerevisiae* as production sites and by improving the geraniol tolerance of the yeast cells. The combination of both approaches led to an 80% increase in the geraniol titers. In the future, the inclusion of product tolerance and peroxisomal compartmentalization into the general chassis engineering toolbox for monoterpenoids or other host-damaging, industrially relevant metabolites may lead to an efficient, low-cost, and eco-friendly microbial production for industrial purposes.

## Introduction

The metabolic engineering of microorganisms such as *Saccharomyces cerevisiae* is a promising tool for the eco-friendly production of aromas, fragrances, biofuels, and pharmaceutics (Zebec et al., 2016). The challenges lie in optimizing the metabolic flux and reducing the toxicity of the desired substances to the production host (Chen et al., 2020). Monoterpenoids, the smallest group of isoprenoids, are of great industrial interest because of their pleasant aroma and odor, and their potential applications as biofuels and pharmaceuticals (George et al., 2015). Geraniol, an acyclic monoterpenoid with a characteristic rose-like odor, is a constituent of plant essential oils like rose, citronella, or palmarosa oil and is widely used as a fragrance material in perfumes, cosmetics or household products. Further, it is commercially used as an insect repellent and natural pest control agent and it exhibits antimicrobial and antitumor properties (Chen and Viljoen, 2010). The industrial production of geraniol exceeds 1000 tons per year (Lapczynski et al., 2008). It is chemically synthesized from pinene or citral and only small amounts are gained from natural resources for the perfume industry by distillation of citronella oil (Surburg and Panten, 2006). The chemical manufacturing processes require multi-step protocols, the use of high temperatures and separation of isomers by fractional distillation. Further, due to seasonal variations and environmental conditions that affect essential oil production and composition, a consistent supply of biomass for the isolation of geraniol from natural resources is not guaranteed. Heterologous production in microorganisms in principle could provide an alternative eco-friendly and potentially cost-effective synthesis of geraniol (Maury et al., 2005). It benefits from energy and resource efficiency and small waste streams. However, so far the production in microorganisms is not industrially relevant, since only small yields are obtained due to low metabolic flux and the toxicity of geraniol to microorganisms such as *Escherichia coli* or *S. cerevisiae* (Liu et al., 2016; Zhao et al., 2016).

**FIGURE 1:**
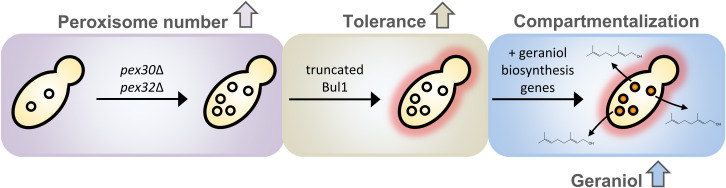
Engineering of tolerant *S. cerevisiae* cells for compartmentalized geraniol production in peroxisomes. Peroxisome number is increased by deleting the genes for the peroxins Pex30 and Pex32. A truncated Bul1 α-arrestin-like adaptor protein, caused by a point-mutation in the corresponding gene, increases geraniol tolerance. Introduction of the geraniol biosynthetic enzymes into the peroxisomes leads to geraniol production.

In this study, we evaluated two approaches for their suitability to be integrated into the engineering toolbox to increase geraniol product titers in *S. cerevisiae* cells for industrial biomanufacturers: increasing the tolerance of yeast cells to geraniol and compartmentalizing the geraniol producing enzymes into peroxisomes as production sites (Figure 1). Compartmentalization of biosynthetic enzymes offers the possibility of increasing the metabolic flux due to spatial proximity and increased local concentration of enzymes, improved substrate channeling and reduced metabolic crosstalk (DeLoache and Dueber, 2013). Peroxisomes, ubiquitous eukaryotic organelles with a single bilayer membrane, have already proven to be suitable compartments for the production of polyhydroxyalkanoates used for biodegradable plastics as well as for the production of fatty alcohols, alkanes, and olefins used as drop-in fuels (Poirier et al., 2001; DeLoache et al., 2016; Zhou et al., 2016). Recently, peroxisomes were successfully used as compartments for the production of the triterpene squalene (Liu et al., 2020). Here, we tested their suitability for monoterpenoid production. In a first step, *S. cerevisiae* cells with constantly high numbers of peroxisomes were generated by deleting the peroxin-encoding genes *PEX30*, *PEX31*, and *PEX32*, involved in peroxisome biogenesis (Vizeacoumar et al., 2004), as well as the pexophagy receptor-encoding gene *ATG36*, involved in peroxisome degradation (Motley et al., 2012a), in all possible combinations. Spot tests revealed that strains lacking the peroxins Pex30 and Pex32 separately and in combination showed an increased tolerance to geraniol. Comparative genomic analysis of these strains identified randomly inserted single-nucleotide polymorphisms (SNPs) resulting in a truncated α-arrestin-like adaptor Bul1, which seems to be responsible for the increase in geraniol tolerance. The double deletion strain *pex30*Δ/*pex32*Δ with high peroxisome numbers and high geraniol tolerance as well as the triple deletion strain *pex30*Δ/*pex31*Δ/*atg36*Δ with high peroxisome numbers but low geraniol tolerance were used for the insertion of the geraniol biosynthetic enzymes into peroxisomes and into cytoplasm as control. Whereas the compartmentalization into peroxisomes increased the geraniol titer by up to 13% in both strain backgrounds compared to cytoplasmic localization, the production was increased by up to 63% in the geraniol tolerant strain background *pex30*Δ/*pex32*Δ compared to the sensitive strain background *pex30*Δ/*pex31*Δ/*atg36*Δ. The combination of both approaches finally led to an 80% increase in geraniol titer in the tolerant strain with peroxisomal enzyme localization. Combined with other established tools to increase the metabolic flux (Zhao et al., 2017), the usage of geraniol tolerant strains and compartmentalization into peroxisomes are promising approaches to elevate geraniol titers to a level acceptable for industrial applications and to push ahead eco-friendly and cost-efficient production of this industrially valuable natural product.

## Results

### Single and Multiple Deletions of *PEX30*, *PEX31*, *PEX32*, and *ATG36* Increase the Peroxisome Number of *Saccharomyces cerevisiae* Cells in Glucose Medium

**FIGURE 2:**
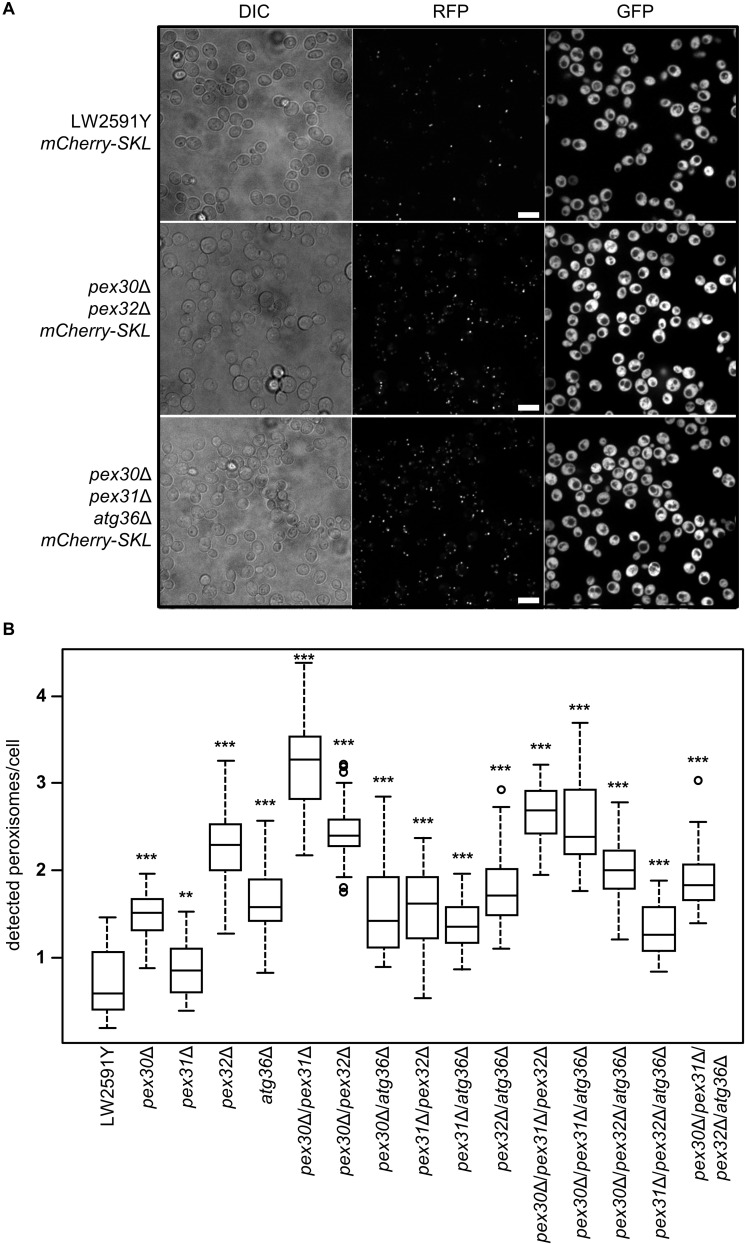
Strains with all possible combinations of *PEX30*, *PEX31*, *PEX32*, and *ATG36* deletions show increased peroxisome numbers. **(A)** Fluorescence microscopy of the reference *S. cerevisiae* strain LW2591Y, *pex30*Δ/*pex32*Δ, and *pex30*Δ/*pex31*Δ/*atg36*Δ with integrated *mCherry-SKL* under a constitutive promoter. Yeast strains were grown overnight in liquid YPD medium at 30°C, diluted and used for microscopy. Due to the reiterative recombination system site in the parental strain, all strains carry *gfp* integrated into the genome. Scale bar = 10 μm. **(B)** Quantification of the number of peroxisomes per cell detected via fluorescence microscopy in the parental strain LW2591Y and all *PEX30*, *PEX31*, *PEX32*, and *ATG36* deletion combinations with introduced mCherry-SKL in YPD medium. For counting of the cells and peroxisomes in total 76 fluorescence images from two biological replicates were taken and the peroxisome/cell ratios were calculated. The 76 ratios are depicted in a Tukey’s box whisker plot. The *p*-value for each strain was calculated in comparison to LW2591Y (two-sample *T*-test). ***p ≤ 0.01; **p ≤ 0.05.

*Saccharomyces cerevisiae* cells strictly regulate the peroxisome number in response to environmental signals by the interplay between biogenesis of new and degradation of present peroxisomes (Sibirny, 2016). At a molecular level, the regulation depends on the function of various *PEX* and *ATG* genes (Supplementary Figure S1). In order to obtain strains with a constant high number of peroxisomes, the genes for the peroxisome number controlling proteins Pex30, Pex31, and Pex32, as well as for the pexophagy receptor Atg36 were deleted in all possible combinations in LW2591Y, resulting in 15 deletion strains (Supplementary Table S1). A gene coding for the red fluorescence protein mCherry with the C-terminal, peroxisomal SKL amino acid target sequence (PTS1) was integrated (Till et al., 2012). The peroxisomes were quantified with fluorescence microscopy after growth in glucose-containing YPD medium (Figure 2A). All 15 deletion strains showed an increase in peroxisome number compared to the parental strain (Figure 2B and Supplementary Figure S2). The highest peroxisome number with a 5.6-fold increase was observed for *pex30*Δ/*pex31*Δ. The lowest number was found for *pex31*Δ with a 1.4-fold increase in comparison to the control.

### Yeast Strains Carrying a Truncated α-Arrestin-Like Adaptor Bul1 Show a Highly Increased Tolerance to Geraniol

**FIGURE 3:**
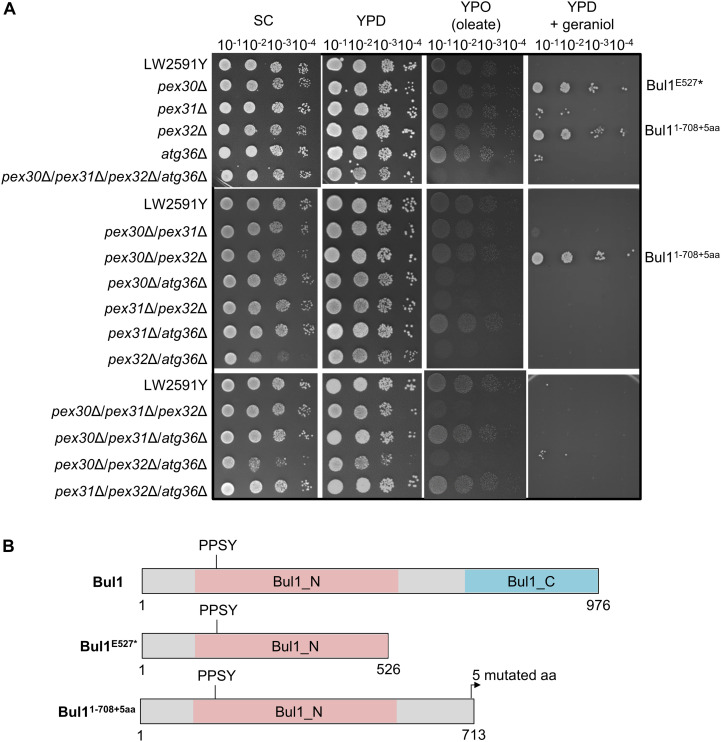
The *S. cerevisiae* strains *pex30*Δ, *pex32*Δ, and *pex30*Δ/*pex32*Δ with a truncated Bul1 protein are highly tolerant to geraniol. **(A)** Spot-tests of the reference strain LW2591Y and all single, double, triple, and quadruple deletion mutants of *PEX30*, *PEX31*, *PEX32*, and *ATG36* on a synthetic complete medium (SC), a yeast extract peptone medium with glucose (YPD) or with oleate (YPO) as sole carbon-source and on YPD with 200 mg/l geraniol. A serial dilution was used (OD = 10^–1^, 10^–2^, 10^–3^, 10^–4^). The plates were incubated at 30°C for 3 days for SC, YPD, and YPD + geraniol and for 6 days for YPO medium. **(B)** Schematic representation of the truncated Bul1 proteins present in the tolerant strains *pex30*Δ, *pex32*Δ, and *pex30*Δ/*pex32*Δ. The wildtype Bul1 harbors an N-terminal conserved domain (Bul1_N) with the ubiquitin ligase binding motif PPSY and a C-terminal conserved domain (Bul1_C). *pex30*Δ carries a SNP from G to T at position 1579 bp of the *BUL1* gene, leading to a stop-codon and a truncated Bul1 protein with 526 aa (Bul1^E527*^). *pex32*Δ carries an additional adenine and *pex30*Δ/*pex32*Δ an additional thymine at position 2125 bp of the *BUL1* gene, leading to a truncated Bul1 protein with 713 aa (Bul1^1–708+5aa^). The nucleotide insertions induce a frameshift, leading to the insertion of an early stop-codon in addition to 5 mutated amino acids at the end of the truncated protein.

Geraniol is toxic for yeast cells and the addition of 200 mg/l geraniol completely inhibits the growth of laboratory reference strains (Zhao et al., 2016). Therefore, the deletion strains were tested for growth deficiencies, which might cause problems when inserting the biosynthesis genes for geraniol. The strains were cultivated on synthetic complete (SC) medium and on yeast extract peptone medium with glucose (YPD) or oleate (YPO) as sole carbon source (Figure 3A). Oleate is metabolized by peroxisomal enzymes and is therefore an indicator for peroxisome functionality (Gurvitz and Rottensteiner, 2006). Whereas the single deletions showed no growth deficiencies on any medium, the double deletion *pex32*Δ/*atg36*Δ showed a diminished growth on SC and the triple deletion *pex30*Δ/*pex32*Δ/*atg36*Δ on SC and YPD. On oleate medium (YPO), the double deletions *pex30*Δ/*atg36*Δ, *pex31*Δ/*pex32*Δ, and *pex32*Δ/*atg36*Δ, the triple deletions *pex30*Δ/*pex31*Δ/*pex32*Δ and *pex30*Δ/*pex32*Δ/*atg36*Δ as well as the quadruple deletion *pex30*Δ/*pex31*Δ/*pex32*Δ/*atg36*Δ were diminished in growth, indicating a reduced functionality of the peroxisomes.

Toxic effects of geraniol were investigated by cultivation of all strains on YPD with 200 mg/l geraniol (Figure 3A). In agreement with the literature (Zhao et al., 2016), the reference strain did not grow. The deletion strains *pex30*Δ, *pex32*Δ, and *pex30*Δ/*pex32*Δ showed a highly increased tolerance to geraniol treatment. *pex31*Δ, *atg36*Δ, *pex30*Δ/*pex31*Δ, and *pex30*Δ/*pex32*Δ/*atg36*Δ showed weak growth, whereas all other mutants did not grow, similar to the reference strain. On plates with 250 mg/l geraniol, the tolerant strains *pex30*Δ, *pex32*Δ, and *pex30*Δ/*pex32*Δ did not grow anymore (Supplementary Figure S3). In order to determine the reason for the high tolerance in *pex30*Δ, *pex32*Δ, and *pex30*Δ/*pex32*Δ compared to the reference strain LW2591Y, a genome comparison was conducted, revealing the accumulation of frame shifts within the *BUL1* gene resulting in premature stop-codons mutants. A SNP analysis of *pex30*Δ/*pex32*Δ showed a thymine insertion at position 2125 in the *BUL1* gene, encoding an α-arrestin-like adaptor protein. The induced frameshift leads to the introduction of an early stop-codon and results in a truncated Bul1 protein with 713 aa missing the C-terminal domain (Figure 3B). Sanger sequencing of the *BUL1* gene of all *PEX*/*ATG* deletion strains revealed that in *pex32*Δ an adenine nucleotide was inserted at the same position as in *pex30*Δ/*pex32*Δ leading to a truncated Bul1 with the same size. In *pex30*Δ, a nucleotide substitution from guanine to thymine at position 1579 bp of *BUL1* leads to the introduction of a stop-codon, resulting in a truncated Bul1 protein with only 526 aa (Figure 3B). A spot-test of a *BUL1* deletion strain in BY4741 background on geraniol-containing YPD medium verified that the loss of an intact Bul1 increases the geraniol tolerance (Supplementary Figure S4). *pex30*Δ and *pex32*Δ in BY4741 background carrying an intact *BUL1* gene showed no increased geraniol tolerance, supporting the result that a loss-of-function Bul1 is responsible for the geraniol tolerance in the *PEX* strains in LW2591Y background.

In order to analyze and compare the influence of geraniol tolerance and compartmentalization into peroxisomes on the geraniol titer, two strains were selected for the introduction of the geraniol biosynthesis pathway that meet the following criteria: (1) one strain with and one strain without increased geraniol tolerance, (2) high peroxisome numbers, and (3) no growth deficiencies. Accordingly, *pex30*Δ/*pex32*Δ with high geraniol tolerance due to the truncated Bul1 protein and *pex30*Δ/*pex31*Δ/*atg36*Δ with low geraniol tolerance were chosen. Peroxisome numbers of both strains were further quantified with fluorescence microscopy as described before on glucose-containing SC medium, which corresponds to the cultivation conditions for geraniol extraction. After one and two days, both strains show a significant increase compared to the reference strain LW2591Y, although the overall peroxisome/cell ratio is reduced compared to YPD medium (Supplementary Figure S5).

### Introduction of Geraniol Biosynthesis Pathway Genes Into Geraniol-Tolerant *pex30*Δ/*pex32*Δ and Geraniol-Sensitive *pex30*Δ/*pex31*Δ/*atg36*Δ Yeast Strains

Geraniol can be metabolized by *S. cerevisiae* into geranyl acetate through acetylation and into citronellol through reduction. Therefore, the genes *ATF1*, encoding an alcohol acetyltransferase, and *OYE2*, encoding an NADPH oxidoreductase, were deleted in *pex30*Δ/*pex32*Δ and *pex30*Δ/*pex31*Δ/*atg36*Δ (Supplementary Figure S6A; Steyer et al., 2013; Brown et al., 2015).

**FIGURE 4:**
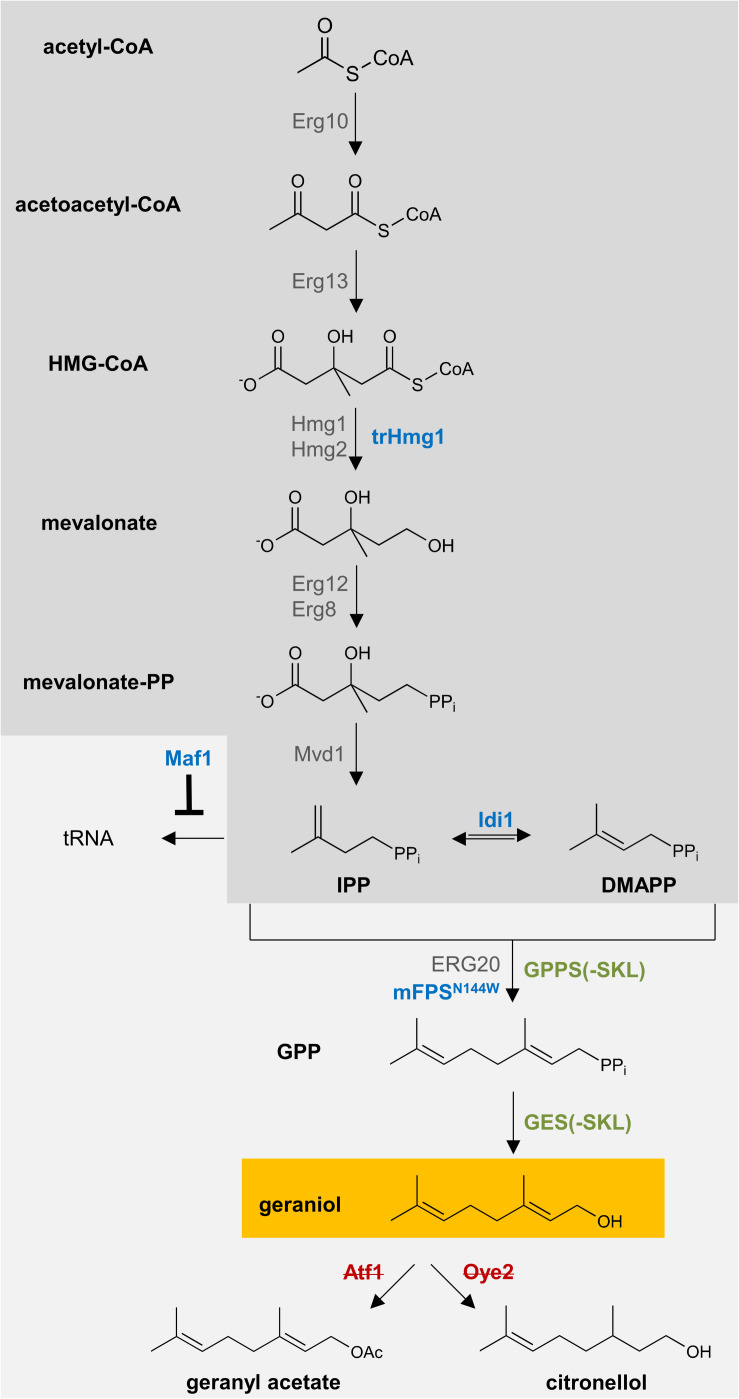
Schematic representation of the engineered biosynthetic pathway for geraniol in *S. cerevisiae*. Geranyl pyrophosphate (GPP) is produced in wildtype *S. cerevisiae* via the mevalonate pathway (dark gray box). One copy of the genes encoding the blue enzymes were introduced into the yeast genome. The genes for the green enzymes were introduced on a high-copy plasmid. For peroxisomal localization of the green enzymes, the peroxisomal target sequence 1 (PTS1), consisting of the amino acids SKL, was attached to the C-terminus. All genes were expressed under constitutive promoters. The genes encoding the red, crossed enzymes have been deleted from the genome.

The biosynthetic precursor of geraniol is geranyl pyrophosphate (GPP). In yeast, GPP is naturally produced via the mevalonate pathway and needed for the production of sterols for the cell membrane (Oswald et al., 2007). To increase the flux through the mevalonate pathway, previously reported and well-established strategies were adapted (Figure 4). The following protein-encoding genes were integrated under the control of strong, constitutive promoters into the *S. cerevisiae* chromosomal genome of *pex30*Δ/*pex32*Δ and *pex30*Δ/*pex31*Δ/*atg36*Δ (Supplementary Figure S6A): an N-terminally truncated 3-hydroxy-3-methylglutaryl-coenzyme A (HMG-CoA) reductase 1 without feedback-regulation (trHMG1) (Donald et al., 1997; Rico et al., 2010); the isopentenyl diphosphate (IPP) isomerase Idi1 (Ignea et al., 2011); the negative regulator of RNA polymerase III Maf1, which represses tRNA biosynthesis from IPP (Liu et al., 2013); and the FPP-synthase from *Gallus gallus* carrying a point mutation for the N144W amino acid substitution (mFPS^*N144W*^), which produces high amounts of GPP (Stanley Fernandez et al., 2000). The resulting strains were named Sen (“Sensitive,” in *pex30*Δ/*pex31*Δ/*atg36*Δ with low geraniol tolerance) and Tol (“Tolerant,” in *pex30*Δ/*pex32*Δ with high geraniol tolerance). We have integrated these genes on the chromosomes to enhance genetic stability. In comparison to an expression of the genes from high copy plasmids, this will most probably lead to reduced gene doses and less expression, which will most likely be accompanied by lower geraniol titers. However, it also leads to a higher comparability between the different strains, since exactly one additional copy of the gene is present in all strains. The expression level of the integrated genes was verified with semiquantitative reverse transcription-PCR (Supplementary Figure S7A). Whereas for *trHMG1*, *MAF1*, and *mFPS*^*N144W*^ the gene expression was increased in Tol and Sen compared to LW2591Y, the expression level of *IDI1* was comparable to LW2591Y in both strains.

Finally, the codon-optimized genes for the GPP-synthase (GPPS) from *Abies grandis* (AgGPPS2) and the geraniol-synthase (GES) from *Ocimum basilicum* were inserted under strong, constitutive promoters on a high copy plasmid in the strains Tol and Sen (Supplementary Figure S6B). The N-terminal chloroplast targeting sequences of both genes were removed. To compare cytoplasmic (Cyt) and peroxisomal (Per) production of geraniol, both genes were inserted with and without the PTS1 motif for peroxisomal localization. The successful expression of the inserted genes was verified with semiquantitative reverse transcription-PCR (Supplementary Figure S7B). As control strain, an empty vector without *GPPS* and *GES* (Neg) was used. The resulting six strains Sen_Cyt, Sen_Per, Sen_Neg, Tol_Cyt, Tol_Per, and Tol_Neg are listed in Table 1 and Supplementary Table S1.

**TABLE 1:** Growth rate μ, maximum cell growth A and absolute geraniol titer of indicated *S. cerevisiae* strains.

Strain	*PEX* and *ATG* gene deletions	Geraniol tolerance	Localization of GPPS/GES	Growth rate μ	Max. cell growth A	Geraniol titer [mg/l]
Sen_Neg	*PEX30*; *PEX31*; *ATG36*	Low	−	0.076 ± 0.003	0.978 ± 0.024	0
Sen_Cyt	*PEX30*; *PEX31*; *ATG36*	Low	Cytoplasm	0.059 ± 0.003	0.822 ± 0.020	1.53 ± 4.8%
Sen_Per	*PEX30*; *PEX31*; *ATG36*	Low	Peroxisomes	0.035 ± 0.005	0.778 ± 0.043	1.68 ± 8.3%
Tol_Neg	*PEX30*; *PEX32*	High	−	0.082 ± 0.007	0.957 ± 0.029	0
Tol_Cyt	*PEX30*; *PEX32*	High	Cytoplasm	0.049 ± 0.005	0.851 ± 0.020	2.44 ± 0.04%
Tol_Per	*PEX30*; *PEX32*	High	Peroxisomes	0.046 ± 0.002	0.854 ± 0.032	2.75 ± 1.3%

*Data for μ and A are from two biological replicates with each three technical replicates and give the mean with standard deviation. Data for geraniol titers are from two biological replicates with each two technical GCMS replicates. Data give the mean with the percentage difference.*

GPP-synthase and geraniol-synthase with their peroxisomal SKL target sequence were both N-terminally tagged with mCherry and analyzed with fluorescence microscopy to examine whether they are accurately located within the cell (Supplementary Figure S8A). Both proteins were found to be localized to the peroxisomes. In case of mCherry-GES-SKL an additional signal in the cytoplasm is visible indicating either an incomplete transport into the peroxisomes or a partially instable mCherry-GES-SKL fusion protein. A Western analysis was performed using an antibody against mCherry (Supplementary Figure S8B). Whereas there is a single signal detectable for mCherry-GPPS-SKL, a band with the correct size is visible for mCherry-GES-SKL plus several smaller bands, indicating degraded protein versions. We assume that the degraded protein bands are responsible for the cytoplasmic signal in the fluorescence microscopy.

### Peroxisomal Localization of GPP-Synthase and Geraniol-Synthase Reduces the Yeast Cell Growth Rate Compared to Cytosolic Localization

**FIGURE 5:**
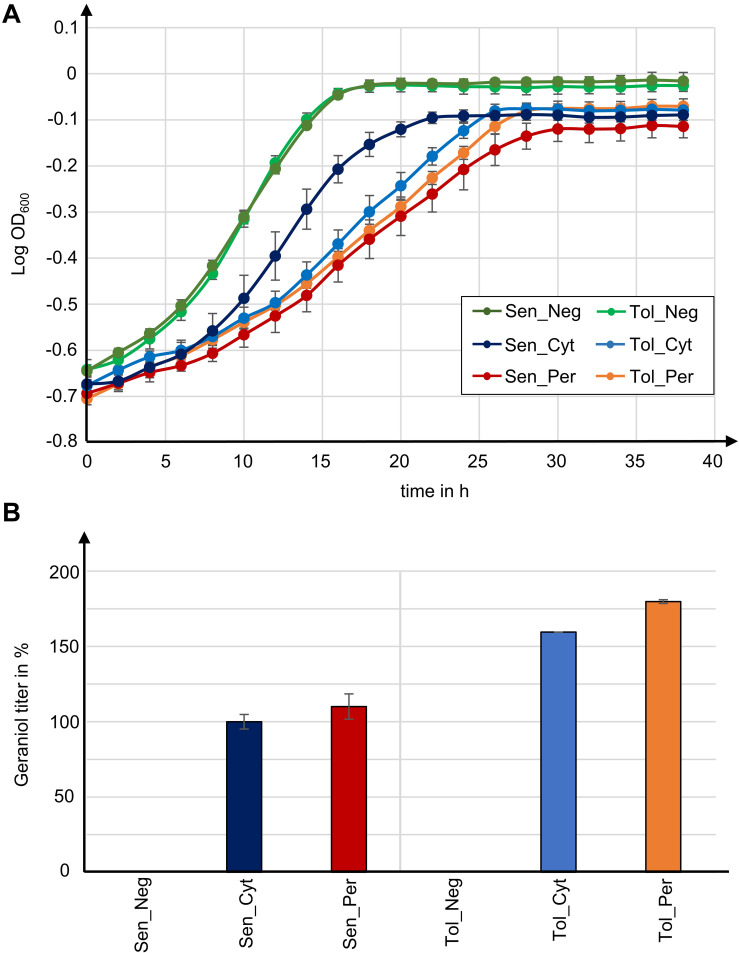
Tolerance to geraniol combined with peroxisomal enzyme localization in yeast strains with high peroxisome numbers yields highest geraniol titer in this study. **(A)** Growth curves for indicated *S. cerevisiae* mutants. Data give the means with standard deviation from two biological with each three technical replicates. **(B)** Relative geraniol titer calculated from absolute values in Table 1. Data give the means with percentage difference from two biological with each two technical GCMS replicates.

Geraniol is toxic for *S. cerevisiae* cells and reduces the growth rate. Growth curves for the geraniol-producing strains Sen_Cyt, Sen_Per, Tol_Cyt, and Tol_Per, as well as for the non-producing control strains Sen_Neg and Tol_Neg were compared (Figure 5A) and the growth rates μ as well as the maximum cell growth A were determined (Table 1).

The two control strains Sen_Neg and Tol_Neg grow in an equivalent manner with similar growth rates of 0.076 and 0.082, respectively, resulting in a similar maximum cell growth. All geraniol producing strains show a general growth deficiency. Growth rates vary greatly in the sensitive strains Sen_Cyt (μ = 0.059) and Sen_Per (μ = 0.035), resulting in the lowest determined maximum cell growths with A = 0.822 for Sen_Cyt and A = 0.778 for Sen_Per. The growth rates of the two tolerant strains Tol_Cyt (μ = 0.049) and Tol_Per (μ = 0.046) are similar, leading to similar maximum cell growth values with A = 0.851 and A = 0.854, respectively.

### Geraniol Tolerance and High Peroxisome Numbers Combined With Peroxisomal Biosynthesis Improve Geraniol Titers up to 80%

For quantification of the geraniol titer, geraniol was extracted from Sen_Neg, Sen_Cyt, Sen_Per, Tol_Neg, Tol_Cyt, and Tol_Per and analyzed by gas chromatography coupled to mass spectrometry (GCMS). All strains except the two control strains with empty vector Sen_Neg and Tol_Neg produced geraniol in detectable amounts (Figure 5B, Table 1, and Supplementary Table S2).

The geraniol sensitive strains Sen_Cyt and Sen_Per produced the overall lowest geraniol levels. Sen_Cyt with cytoplasmic biosynthesis produced 1.53 mg/l geraniol (100%), whereas peroxisomal biosynthesis increased the geraniol titer to 110% in Sen_Per yielding 1.68 mg/l. The geraniol-tolerant strains Tol_Cyt and Tol_Per have an overall elevated geraniol production. In both strains the levels are increased to 159–163% compared to the corresponding Sen_Cyt and Sen_Per strains with 2.44 mg/l in Tol_Cyt and 2.75 mg/l in Tol_Per. Similar to Sen_Cyt and Sen_Per, the geraniol yield is increased to 113% when synthesized in the peroxisomes compared to the cytoplasm. The comparison of Sen_Cyt with 1.53 mg/l and Tol_Per with 2.75 mg/l reveals that the combination of peroxisomal biosynthesis with the usage of geraniol tolerant strains increased the geraniol titer by 80%.

In order to check whether the geraniol titer increase in strains with peroxisomal enzyme localization depends on the number of peroxisomes, we have inserted *GPPS* and *GES* with and without the SKL tag as well as the mevalonate pathway genes *trHMG1*, *IDI1*, *MAF1*, and *mFPS*^*N144W*^ in the parental strain LW2591Y, which has a low number of peroxisomes (Figure 2B and Supplementary Figures S2, S5). The resulting strains LW2591Y_Cyt (cytoplasmic GPPS and GES) and LW2591_Per (peroxisomal GPPS-SKL and GES-SKL) produced 1.47 and 0.95 mg/l, respectively (Supplementary Figure S9). This decrease between cytoplasmic and peroxisomal enzyme localization by 35% leads to the assumption that the increased geraniol titers in strains Tol_Per and Cyt_Per with peroxisomal enzyme localization depend on the number of peroxisomes.

Taken together, these results show that in the presence of high peroxisome numbers (i) strains with high geraniol tolerance show an up to 63% increased geraniol production compared to sensitive strains, (ii) peroxisomal biosynthesis increases the geraniol yield up to 13% compared to cytoplasmic production, and (iii) the combination of both approaches increased the geraniol titer by 80%.

## Discussion

Geraniol is a highly valuable fragrance material for the cosmetics industry with further useful applications (Chen and Viljoen, 2010). Whereas the industrial chemical synthesis is energy consuming, its extraction from plants suffers from low yields and seasonal variations (Surburg and Panten, 2006). For an eco-friendly production with stable yields and probably low costs, the production of geraniol in microorganisms is an optimal solution (Maury et al., 2005). That said and despite much research effort, monoterpenoid production in microorganisms is limited by low metabolic flux through engineered monoterpenoid production pathways and product toxicity (Shah et al., 2013; Zhao et al., 2016). To circumvent both effects, this study applied compartmentalization of biosynthetic enzymes into peroxisomes in tolerant strains using geraniol production as an example, resulting in an 80% increase of product titers. For several years, peroxisomes have been considered as compartments for heterologous metabolic pathways (DeLoache et al., 2016). Peroxisomes are also naturally used as metabolite production sites, e.g., for the penicillin production in *Aspergillus nidulans* or for the AK-toxin biosynthesis in *Alternaria alternata* (Imazaki et al., 2010; Herr and Fischer, 2014), and increasing the number of peroxisomes can increase metabolite titer (Herr and Fischer, 2014). Since AK-toxin is a mevalonate pathway-derived terpenoid, the peroxisomal environment seems to be adequate for the biosynthesis of the monoterpenoid geraniol.

Yeast peroxisomes are generated and degraded depending on nutritional conditions (Sibirny, 2016). On glucose medium, low numbers of peroxisomes are present in a *S. cerevisiae* cell and excessive peroxisomes are degraded via an autophagic process called pexophagy coordinated by the specific pexophagy receptor Atg36 (Motley et al., 2012a,b; Till et al., 2012). In contrast, on oleate-containing medium, high numbers of peroxisomes are generated via fission or *de novo* proliferation from the endoplasmic reticulum depending on Pex30, Pex31, and Pex32 amongst others (Nuttall et al., 2011; Joshi et al., 2016). For the industrial production of geraniol or other compounds inside peroxisomes, constantly high peroxisome numbers as well as cultivation in cheap glucose medium would be beneficial. A previous study showed that *PEX30*, *PEX31*, and *PEX32* deletions in different combinations increased the average number of peroxisomes per cell on oleate medium (Vizeacoumar et al., 2004). Here, we investigated peroxisome numbers during growth on glucose medium (YPD) and in combination with an *ATG36* deletion (Figure 2B). In general, we confirmed the increase in peroxisome numbers in strains lacking Pex30, Pex31, or Pex32, but the amount differs between cells grown on oleate or glucose as carbon source. Whereas on oleate medium *pex30*Δ/*pex31*Δ/*pex32*Δ showed the highest peroxisome number (Vizeacoumar et al., 2004), on glucose medium the highest increase was achieved when simultaneously deleting *PEX30* and *PEX31* (Figure 2B). The additional deletion of *ATG36* in most of the *PEX* deletions did not lead to an additional increase in peroxisome number but to a decrease compared to the *PEX* deletions alone (Figure 2B). A comparison of the peroxisome functionality of the *PEX*/*ATG* deletion library by spotting on oleate-containing medium revealed that 6 out of 15 deletion mutants showed defects, whereas all single deletions grow normally (Figure 3A), which is in accordance with the literature (Vizeacoumar et al., 2004). For *pex31*Δ/*pex32*Δ it was shown earlier that the localization of the GFP-tagged peroxisomal membrane protein Pex3 was altered, indicating that the protein import machinery of the peroxisome is not working properly and important enzymes for the function of the peroxisomes are not imported correctly (Zhou et al., 2016). This is in line with the observed growth defects on oleate-containing medium for *pex31*Δ/*pex32*Δ. However, mCherry-SKL was correctly imported in all *PEX*/*ATG* deletion strains in fluorescence microscopy for peroxisome quantification. Growth on glucose-containing SC medium led to a general decrease of peroxisomes numbers compared to geraniol-containing YPD medium (Supplementary Figure S5). Still, the deletion strains *pex30*Δ/*pex32*Δ and *pex30*Δ/*pex31*Δ/*atg36*Δ showed an increased peroxisome number compared to the parental strain LW2591Y, but the increase was not as high as for YPD medium (Figure 2B). Thus, the cultivation of strains in YPD medium would be preferable to obtain maximum peroxisome numbers. Since the *GPPS* and *GES* were expressed from plasmids, SC medium without uracil as selection marker was chosen to prevent plasmid loss.

A major drawback in engineering microorganisms as production platforms for monoterpenoids such as geraniol is the toxicity of them to the cells. At a concentration of 200 mg/l geraniol, growth of the laboratory strain CEN.PK102-5B is completely inhibited (Zhao et al., 2016). This is in line with our laboratory strains (Figure 3A and Supplementary Figure S4). The reason for its toxicity might be the membrane-disruptive features of geraniol (Bard et al., 1988; Carnesecchi et al., 2002). One way to circumvent toxicity is to increase the tolerance of the strains. High tolerance was achieved by SNPs in the *BUL1* gene leading to truncated protein variants in *pex30*Δ, *pex32*Δ, and *pex30*Δ/*pex32*Δ (Figure 3A). Bul1 is an α-arrestin-like adaptor that links the E3 ubiquitin-ligase Rsp5 to its substrate for ubiquitination. This process is needed during endocytosis of plasma membrane transporters to adapt to changing external cues (Novoselova et al., 2012). Up to date, two ubiquitination targets of the Bul1/Rsp5 complex are identified: the monocarboxylate transmembrane transporter Jen1 and the general amino acid permease Gap1 (O’Donnell, 2012). Growth on either a preferred nitrogen source such as ammonium or the carbon source glucose induces Bul1/Rsp5-mediated ubiquitination of Jen1 or Gap1, respectively, and stimulates transporter inactivation via endocytosis. Consequently, a non-functional Bul1 protein prevents transporter degradation and might lead to increased tolerances to chemical compounds. Indeed, the *BUL1* deletion strain is not only tolerant to geraniol, but also to a broad spectrum of other chemicals such as cisplatin, fluorine-containing anesthetics, camptothecin, tunicamycin, bleomycin, or rapamycin (Wolfe et al., 1999; Huang et al., 2005; Kapitzky et al., 2010). Since the truncated Bul1 strains as well as the deletion strain show no fitness defects on glucose medium but a high tolerance to geraniol (Figure 3A and Supplementary Figure S4), the non-functional Bul1 is a promising candidate for being incorporated as integral part into the geraniol engineering toolbox in yeast. Here, we gave the proof-of-principle and increased the geraniol titer by 63% in a strain with a Bul1 truncation (Figure 5B). Because of its manifold tolerances to chemicals, *BUL1* deletions might also be considered for improving the heterologous production of other valuable chemical products in yeast. The reason for the weak geraniol tolerance in *pex31*Δ, *atg36*Δ, *pex30*Δ/*pex31*Δ, and *pex30*Δ/*pex32*Δ/*atg36*Δ could not be identified yet (Figure 3A). The genes for *BUL1* were intact in these strains. Possibly, another not yet identified SNP is responsible for the increased tolerance. The future identification of this putative SNP might lead to the identification of a new candidate for further increasing geraniol tolerance in yeast.

For the compartmentalization of enzymes, a suitable targeting signal is necessary for the correct transport into the organelle. We have chosen the PTS1 for peroxisomal localization consisting of the three amino acids SKL attached to the C-terminus of the protein. There are three different common tags for peroxisomal localization. The SKL and the KANL tags are C-terminal PTS1 tags and bind the PEX5 receptor, whereas the N-terminal PTS2 tag binds the Pex7 receptor (Ehrenworth et al., 2017). Among those three tags, the SKL tag was shown to have the highest efficiency with 99%, which is almost independent of plasmid copy number (Ehrenworth et al., 2017). To verify the correct import of the SKL-tagged GPPS and GES in our study, we have tagged the proteins with an N-terminal mCherry protein and analyzed the localization with fluorescence microscopy (Supplementary Figure S8A). Both proteins were visible in the peroxisomes. For mCherry-GES-SKL, an additional signal in the cytoplasm was visible, which was assigned to degraded protein versions without the C-terminal SKL tag (Supplementary Figure S8B).

Compartmentalization of enzymes in general benefits from spatial proximity and increased local concentration of enzymes, improved substrate channeling and reduced metabolic crosstalk (DeLoache and Dueber, 2013). In our study, a 13% increase in geraniol production was observed when inserting the GPPS and GES into the peroxisomes in strains with high peroxisome numbers. In a control strain with low peroxisome numbers, the geraniol titer was decreased in strains with peroxisomal enzymes compared to cytosolic enzymes (Supplementary Figure S9). Although the increase of 13% in strains with high peroxisomes is small and other factors such as the different genetic background caused by the different *PEX*/*ATG* deletions must be considered, a trend can be seen that compartmentalization into peroxisomes is a promising approach to increase geraniol titers. For further optimizations of the titers, the whole MVA pathway can be introduced into the peroxisomes to further improve spatial proximity and local concentrations of precursors as described previously (Liu et al., 2020). The aim of this study was to test new methods for their suitability to be included in the engineering toolbox for geraniol production in yeast. Compared with the literature, our obtained titers are low. We have accepted the low titers in order to achieve a better comparability of the strains. Instead of overexpressing the genes for mevalonate pathway flux optimization on plasmids with several copies per cell, we have integrated only one copy of each gene in a defined spot on the chromosomes. This enables an even expression level and increases comparability between the strains, but the expression levels especially for *IDI1* and *HMG1* were low (Supplementary Figure S7A). The highest geraniol titer obtained from *S. cerevisiae* cultures is reported as 1.69 g/l (Zhao et al., 2017). This was achieved by combined deletion of the NADPH oxidoreductase gene *OYE2* to reduce endogenous geraniol metabolism coupled with control of the GPP flux distribution by dynamic control of the FPP-synthase gene *ERG20* in a leucine prototrophic strain. The strain, which carries the *Valeriana officinalis* GES, was cultivated with pure ethanol feeding in fed-batch fermentation (Zhao et al., 2017). Combinations of these previous approaches with the peroxisomal compartmentalization of geraniol biosynthetic enzymes contained in geraniol-tolerant *S. cerevisiae* strains might further enhance geraniol production. A comparison of the growth rates showed that strains with peroxisomal enzyme localization are reduced compared to the strains with cytosolic enzymes (Figure 5A and Table 1), which might be less optimal for industrial applications. Nevertheless, the tolerant strain with peroxisomal enzymes Tol_Per reaches a similar maximum cell growth after 30 h as the other strains with cytoplasmic enzymes and produced the highest geraniol titer in our study. Titers likely depend on overall available peroxisomal space (Herr and Fischer, 2014). Further increases in production might be reached using the methylotrophic yeasts *Pichia pastoris* and *Hansenula polymorpha* as both have giant peroxisomes that can account for more than 80% of the total cell volume (Veenhuis et al., 1978; Van Der Klei et al., 1991). These general chassis engineering approaches could be extended to other monoterpenoids many of which are important fragrances, flavors, and next-generation biofuels. Compartmentalization in product-tolerant strains will work alongside other strategies to increase product titers to industry production levels. Our work establishes that metabolic compartmentalization and, especially, the usage of product-tolerant strains are important strategies that should be considered for inclusion in the chassis engineering toolbox to increase product titers for industrial biomanufacture.

## Materials and Methods

### Growth Media and Growth Conditions

*Saccharomyces cerevisiae* strains were cultivated on either synthetic complete medium [SC; 0.2% yeast nitrogen base w/o amino acids and ammonium sulfate, 0.5% ammonium sulfate, 2% glucose, 5% (v/v) amino acid mix, and the appropriate supplements 0.002% histidine, 0.002% tryptophan, 0.002% uracil, 0.009% leucine], yeast extract peptone dextrose medium (YPD; 1% yeast extract, 2% bacto peptone, and 1% glucose), or an oleate-containing yeast extract peptone medium [YPO; 0.3% yeast extract, 0.5% bacto peptone, 0.12% (v/v) oleic acid, 0.2% (v/v) Tween-40, 0.5% KH_2_PO_4_ (pH = 6)]. The amino acid mix contains 0.04% adenine sulfate, 0.04% arginine-HCl, 0.2% aspartic acid, 0.2% glutamic acid, 0.06% isoleucine, 0.06% lysine-HCl, 0.04% methionine, 0.1% phenylalanine, 0.8% serine, 0.4% threonine, 0.06% tyrosine, and 0.3% valine. For recycling of the *URA3*-marker 0.05% 5-fluoroorotic acid was used. For solid plates 2% agar was used. For geraniol toxicity tests, 200 mg/l geraniol was added to the YPD medium.

*Escherichia coli* strains were cultivated on lysogeny broth medium (LB; 1% tryptone, 0.5% yeast extract, and 1% NaCl) supplemented with 100 μg/ml ampicillin.

### Codon-Optimization and Synthesis of Genes

For gene synthesis, a geranyl diphosphate-synthase of *Abies grandis* (GenBank: AAN01134.1) without the chloroplast targeting sequence and with a C-terminal SKL-tag, a gene encoding a geraniol-synthase of *Ocimum basilicum* (UniProtKB/Swiss-Prot: Q6USK1.1) without the chloroplast targeting sequence and with a C-terminal SKL-tag, and a gene encoding the farnesyl pyrophosphate-synthase of *Gallus gallus* (UniProtKB/Swiss-Prot: P08836.2) with a N144W mutation (mFPS^N144W^) were chosen. All genes were codon-optimized for *S. cerevisiae* with the GeneOptimizer^TM^ Software, synthesized and cloned with *Pme*I restriction sites into vector pMA-RQ (Thermo Fisher Scientific GENEART GmbH, Regensburg), resulting in plasmids pME4804–4806. In addition, the *HIS3* promoter and terminator, the *CYC1* promoter and terminator, and the *TEF1* promoter and terminator were synthesized and cloned with *Sfi*I restriction sites into the vectors pME4805, pME4806, and pME4804, respectively.

### Plasmids Construction

All used and constructed plasmids are listed in Supplementary Table S3. All used primer sequences are given in Supplementary Table S4. *E. coli* strain DH5α [F^–^, Φ80Δ(*lacZ*)M15^–1^, Δ(*lacZYA-argF*) U169, *recA1*, *endA1*, *hsdR17* (r_K_^–^, m_K_^+^), *supE44*, λ^–^, *thi1*, *gyrA96*, *relA1*] was used (Woodcock et al., 1989).

For targeting a protein to the peroxisomes, the peroxisomal target sequence 1 (PTS1) consisting of the amino acids SKL was fused to the C-terminus of the protein. For this, the corresponding coding sequence was attached to the reverse primer of the gene of interest.

For the construction of pME4803, P*_*GPD1*_* (amplified with JG965/1062 from pME4093), *mCherry*-*SKL* (amplified with JG1063/1064 from pME3772), and T*_*CYC1*_* (amplified with JG1065/969 from pME2787) were assembled together with pBluescript II KS (+) digested with *EcoR*V via the GeneArt^TM^ Seamless Cloning and Assembly Enzyme Mix Kit (Thermo Fisher Scientific).

For pME4804, the *TEF1* promoter, *mFPS*^*N144W*^, and the *TEF1* terminator were synthesized and cloned into the pMA-RQ vector with additional *Sfi*I sites (Thermo Fisher Scientific). In the same manner P*_*HIS3*_*, *GES*-*SKL*, and T*_*HIS3*_* (pME4805) as well as P*_*CYC1*_*, *GPPS*-*SKL*, and T*_*CYC1*_* (pME4806) were designed (Thermo Fisher Scientific).

For pME4807, the *URA3* promoter and terminator were amplified from pME2787 with primers JG1228/1230 and JG1231/1229, respectively. The truncated *HMG1* gene (*trHMG1*) was amplified with primers JG1232 and JG1233 from genomic DNA of LW2591Y. All three fragments and the *EcoR*V digested pBluescript II KS (+) vector were assembled with the GeneArt^TM^ Seamless Cloning and Assembly Kit (Thermo Fisher Scientific) resulting in plasmid pME4807.

For pME4808, the *IDI1* gene was amplified with primers JG1224 and JG1225 from genomic DNA of LW2591Y and cloned with the GeneArt^TM^ Seamless Cloning and Assembly Kit (Thermo Fisher Scientific) into pME4806 digested with *Pme*I, resulting in pME4808, containing the *IDI1* gene with the *CYC1* promoter and terminator.

For pME4809, the *MAF1* gene was amplified with primers JG1226 and JG1227 from genomic DNA of LW2591Y and cloned with the GeneArt^TM^ Seamless Cloning and Assembly Kit (Thermo Fisher Scientific) into pME4805 digested with *Pme*I, resulting in pME4809, containing the *MAF1* gene with the *HIS3* promoter and terminator.

For pME4810, P*_*GPD1*_* (amplified with JG1355/1172 from pME4803), *GPPS*-*SKL* (amplified with JG1220/1221 from pME4806), and T*_*CYC1*_* (amplified with 1137/1346 from pME4803) were assembled together with *Pvu*II digested pESC_URA via the GeneArt^TM^ Seamless Cloning and Assembly Enzyme Mix Kit (Thermo Fisher Scientific). The plasmid carries an additional *Pme*I site downstream of T*_*CYC1*_*. P*_*GPD1*_*, *GPPS* (amplified with JG1220/1234 from pME4806), and T*_*CYC1*_* were assembled in the same way, resulting in pME4811.

For pME4812, P*_*TEF1*_* was amplified with JG1384/1385 from pME4804, *GES*-*SKL* was amplified with JG1269/1389 from pME4805, and T*_*TEF1*_* was amplified with JG1390/1388 from pME4804. All three fragments were assembled with pME4810 digested with *Pme*I via the GeneArt^TM^ Seamless Cloning and Assembly Enzyme Mix Kit (Thermo Fisher Scientific). In the same way P*_*TEF1*_*, *GES* (amplified with JG1269/1386 from pME4805), and T*_*TEF1*_* were assembled with pME4811 digested with *Pme*I, resulting in pME4813 (Supplementary Figure S6B).

For pME4814, pESC_URA was digested with *Pvu*II and the band with a size of 5.3 kb was religated with T4 DNA ligase (Thermo Fisher Scientific).

For pME5088, P*_*GPD1*_*-*mCherry* (amplified with JG965/1811 from pME4803), and *GPPS*-*SKL*-T*_*CYC1*_* (amplified with JG1913/969 from pME4812) were assembled with pBluescript II KS (+) digested with *EcoR*V via the GeneArt^TM^ Seamless Cloning and Assembly Kit (Thermo Fisher Scientific).

For pME5089, *mCherry* was amplified with JG1910/1811 from pME4803 and *GES*-*SKL* was amplified with JG1911/1912 from pME4812. pME4804 was digested with *Pme*I and *mCherry* and *GES*-*SKL* were cloned between P*_*TEF1*_* and T*_*TEF1*_* via the GeneArt^TM^ Seamless Cloning and Assembly Kit (Thermo Fisher Scientific).

### *Saccharomyces cerevisiae* Strain Construction

*Saccharomyces cerevisiae* strain LW2591Y [reiterative recombination parental acceptor strain; *MAT-a inc*; *his3*Δ*200*; *leu2*Δ, *met15*Δ, *trp1*Δ*63*, *ura3*Δ, P*_*PYK*_*-*GFP*-*HIS3*-(HO cleavage site)] (Wingler and Cornish, 2011) was used as background strain. *S. cerevisiae* strains generated and used are listed in Supplementary Table S1.

Gene deletion was performed seamlessly according to Akada et al. (2006). Primers are listed in Supplementary Tables S4, S5. The 0.79 kb long 5′-region of the gene of interest was amplified from genomic DNA of LW2591Y. Additionally, the URA-marker was amplified from plasmid pME2787 with a forward primer, containing a 40 nt overhang matching the 3′-region of the gene of interest plus a minimum of 24 nt overhang matching the 5′-region of the gene of interest, and a reverse primer, containing a 40 nt overhang matching the 3′-region of the gene of interest. Both amplicons were fused via fusion-PCR (Szewczyk et al., 2007), resulting in a PCR-fragment, which can be used directly for transformation. For transformation, the protocol described in Wingler and Cornish (2011) was used.

For the introduction of P*_*GPD1*_*-*mCherry-SKL*-T*_*CYC1*_*, P*_*GPD1*_*-*mCherry-GPPS-SKL*-T*_*CYC1*_*, P*_*TEF1*_*-*mCherry-GES-SKL*-T*_*TEF1*_*, P*_*URA3*_*-*trHMG1*-T*_*URA3*_*, P*_*CYC1*_*-*IDI1*-T*_*CYC1*_*, P*_*HIS3*_*-*MAF1*-T*_*HIS3*_*, and P*_*TEF1*_*-*mFPS*^*N144W*^-T*_*TEF1*_* into the genome, the reiterative recombination system (Wingler and Cornish, 2011) was used. In a first step, the PCR-products for the transformation were amplified. For this, two PCRs with the primers and templates given in Supplementary Table S6 were performed. The template for PCR 2 is the amplicon of PCR 1. The amplicon of PCR 2 can be directly used for transformation together with the corresponding linearized plasmid also given in Supplementary Table S6. The transformation via electroporation, the induction, the selection, and the reiteration were performed according to the protocol described in Wingler and Cornish (2011).

### SNP Analysis of Geraniol-Tolerant *pex30*Δ/*pex32*Δ Strain

Genomic DNA was extracted from overnight cultures of LW2591Y and *pex30*Δ/*pex32*Δ grown in YPD medium. The cells were lysed in 500 μl lysis buffer (30 mM Tris-HCl, 25.5 mM Na_2_-EDTA, 0.5% SDS, pH = 8) with glass beads by shaking for 15 min. Proteins and cell debris were removed by the addition of 100 μl 8M potassium acetate and centrifugation for 15 min at 13,000 rpm. The DNA in the supernatant was precipitated with 300 μl isopropanol. After centrifugation at 13,000 rpm for 15 min and removal of the supernatant, the DNA was resuspended in 400 μl water with 3 μl RNAse A (10 mg/ml) and incubated at 37°C for 10 min. DNA was precipitated with 1 ml ethanol and 10 μl 4 M ammonium acetate, washed with ethanol, dried at 65°C and dissolved in 50 μl water at 65°C. For additional DNA purification, the DNeasy PowerClean Pro Cleanup Kit (Qiagen) was used according to the manufacturer’s instructions, if necessary. Concentration and purity of the isolated DNA was first checked with a Nanodrop ND-1000 (PeqLab) and exact concentration was determined using the Qubit^TM^ dsDNA HS Assay Kit as recommended by the manufacturer (Thermo Fisher Scientific). Illumina shotgun libraries were prepared using the NEBNext Ultra II DNA Library Prep with Beads (New England Biolabs) following the manufacturer’s protocol. To assess quality and size of the libraries, samples were run on an Agilent Bioanalyzer 2100 using an Agilent High Sensitivity DNA Kit as recommended by the manufacturer (Agilent Technologies). Concentrations of the libraries were determined using the Qubit^TM^ dsDNA HS Assay Kit as recommended by the manufacturer (Thermo Fisher Scientific). Sequencing was performed on a MiSeq system with the reagent kit v3 with 600 cycles (Illumina) as recommended by the manufacturer resulting in 6,729,204 paired-end reads for LW2591Y and 9,020,510 paired-end reads for RH3716 with average read lengths 260 and 269 bp, respectively. Reads were quality filtered with fastp v0.20.0 (Chen et al., 2018) with including overlap correction, removal of sequences with qualities <20, soft clipping with sliding window of 4, removal of sequences <50 bp, and Illumina adapter removal. Quality filtering resulted in 6,525,536 and 8,709,042 paired-end reads with average read lengths 257 and 267 bp. In addition, potential adapter remains were removed with cutadapt v2.5 (Martin, 2011) and potential phiX contamination was removed with bowtie2 v2.3.5.1 (Langmead and Salzberg, 2012) employing default parameters. SNP analysis of LW2591Y and RH3716 was performed with breseq v0.35.0 (Deatherage and Barrick, 2014) against the reference strain S288C (GCA_000146045.2) with default parameters. Sequence data were deposited in the NCBI Sequence Read Archive under the accession numbers SRR11285961 (RH3716) and SRR11285962 (LW2591Y) under Bioproject PRJNA611915.

### Sanger Sequencing of *PEX*/*ATG* Deletion Strains

Genomic DNA of the *PEX*/*ATG* deletion strains was extracted according to the protocol described for SNP analysis and served as template for a PCR with primers JG1882/1883 and Phusion^TM^ DNA Polymerase (Thermo Fisher Scientific) to amplify the *BUL1* gene. The amplified DNA was subjected to Sanger sequencing with primers JG1874 and JG1885 at Microsynth AG in Göttingen, Germany.

### Semiquantitative Reverse Transcription-PCR

The strains were inoculated in 50 ml SC medium to an OD_600_ = 0.1 from an overnight culture and grown at 30°C to an OD_600_ ≈ 0.6. The cells were pelleted, washed, ground with liquid nitrogen and RNA was extracted with the RNeasy Plant Mini Kit (Qiagen). DNase digestion and subsequent cDNA synthesis with random hexamers was carried out using the QuantiTect Reverse Transcription Kit (Qiagen) on 0.8 mg of total RNA for each sample. Amplification was performed with the Phusion^TM^ High-Fidelity DNA Polymerase (Thermo Fisher Scientific) using 1 μl of cDNA and the following primers: JG1660/1233 for *trHMG1*, JG1659/1656 for *IDI1*, JG1301/1658 for *MAF1*, JG1304/1720 for *mFPS*^*N144W*^, JG1515/1318 for *GPPS*, and JG1357/1320 for *GES* (Supplementary Table S4). Amplification conditions were as follows: 40 s at 98°C, 25 cycles of 15 s at 98°C, 30 s at 60°C, and 12 s at 72°C with an adjacent step at 72°C for 5 min. The amplicons were separated on a 1% agarose gel with the Thermo Scientific^TM^ GeneRuler^TM^ 1 kb DNA ladder SM0311 (Thermo Fisher Scientific) as size marker.

### Peroxisome Quantification With Fluorescence Microscopy

The strains were grown for 1 or 2 days in liquid YPD or SC medium at 30°C, diluted approximately 1:10 with water and directly used for fluorescence microscopy. Per strain 76 (for YPD medium) and 60 (for SC medium) fluorescent images (38 and 20 per replicate, respectively) with 100× magnification were randomly taken using a Z1 microscope (Zeiss) equipped with a CSU-X1 A1 confocal scanner unit (Yokogawa), QuantEM:512SC digital camera (Photometrics) and SlideBook 6.0 software package (Intelligent Imaging Innovations). The number of cells (green fluorescence, GFP) and peroxisomes (red fluorescence, mCherry-SKL) were automatically counted from each image with the SlideBook 6.0 software package. The ratio of peroxisomes/cell was calculated from the number of cells and the number of peroxisomes per image, resulting in 76 and 60 values per strain, respectively. The values were depicted in a Tukey’s box-whisker-plot using R (R Development Core Team, 2011) and ggplot2 (Wickham, 2016).

The coding gene of GFP, which fluorescence was used for counting the cells, was integrated into the genome of the *S. cerevisiae* strains during the process of reiterative recombination (Supplementary Figure S6A; Wingler and Cornish, 2011). The fluorescence of mCherry, which was tagged with the peroxisomal target sequence 1, SKL, was used for counting the peroxisomes. In total, peroxisomes of a minimum of 2,131 cells per strain from two biological replicates for YPD medium and a minimum of 2,886 cells per strain from three biological replicates for SC medium were quantified. For statistical analysis and calculation of *p*-values, the two-tailed two-sample *T*-test was used.

### Fluorescence Microscopy for Protein Localizations

The strains were grown overnight in liquid SC medium at 30°C, diluted approximately 1:10 with water and directly used for fluorescence microscopy. Images were taken with 100× magnification using a Z1 microscope (Zeiss) equipped with a CSU-X1 A1 confocal scanner unit (Yokogawa), QuantEM:512SC digital camera (Photometrics) and SlideBook 6.0 software package (Intelligent Imaging Innovations).

### Western Blot Analysis

Forty microgram protein extract was separated by SDS-PAGE and transferred to a nitrocellulose membrane by electroblotting as described previously (Opitz et al., 2017). Total proteins were visualized with Ponceau Red (0.2% Ponceau S, 3% trichloroacetic acid). The membrane was blocked with Tris-buffered saline with Tween20 (TBS-T) with 5% skim milk powder for 1 h. α-RFP antibody (Abcam, ab62341), diluted 1:500 in blocking buffer, was used as primary and goat anti-rabbit IgG horseradish peroxidase conjugate (MoBiTec, G21234), diluted 1:1000 in blocking buffer, as secondary antibody.

### Growth Tests

For growth tests on solid medium, the strains were grown to mid-log phase and diluted to an OD_600_ of 0.1. After preparing serial 10-fold dilutions, the cells were spotted on SC medium, YPD medium, YPO medium or YPD containing geraniol, respectively. The plates were incubated for 3 days at 30°C. The experiments were repeated for at least two biological replicates (*n* = 2) with each three technical replicates. For growth tests in liquid medium, the strains were inoculated from an overnight culture to an OD_600_ = 0.2 in 200 μl SC medium without histidine and uracil. Optical density measurements at 600 nm were performed in two biological (*n* = 2) and three technical replicates in 96-well plates for 40 h using a microplate reader with temperature control at 30°C (Infinite M200, Tecan) and the *Magellan 7.2 software (Tecan)*. The growth rate μ and the maximum cell growth A were calculated using the grofit package for R (Kahm et al., 2010; R Development Core Team, 2011) and the spline fit. The two-tailed two-sample *T*-test was used to calculate the *p*-values.

### Geraniol Extraction and Quantification With Gas Chromatography-Mass Spectrometry (GC-MS)

The strains were inoculated from an overnight liquid culture in 5 ml SC medium without histidine and uracil to an OD_600_ = 0.1. The strains were cultivated for 2 days at 30°C with constant shaking in two biological replicates (*n* = 2). The OD_600_ was measured and the culture was extracted with 1 ml dodecane. The dodecane phase was dried with MgSO_4_. 200 μl of the dodecane phase were mixed with 4 μl 0.1% (w/v) menthol in dodecane as an internal standard and measured in a gas chromatography-mass spectrometry (GC-MS) system from Thermo Fisher Scientific, consisting of a TRACE GC Ultra gas chromatograph and a DSQ Quadrupole mass spectrometer with two technical replicates. The system was equipped with a TRI PLUS RSH autoinjector and a DB-WAX Ultra Inert column (30 m × 0.25 mm, 0.25 μm, Agilent Technologies). Samples were injected with a split ratio of 1:10 and helium as carrier gas at a flow rate of 1 ml/min. The PTV injector temperature was 250°C. The oven temperature gradient program started at 40°C for 1 min followed by heating to 230°C with a rate of 15°C/min and a hold for 3 min. The column effluent was introduced into the EI(+) ion source (200°C) of the MS. The mass scan range was m/z 50–300 (0.52 s). Geraniol was quantified using linear calibration curves from 5 × 10^–5^% (w/v) to 0.01% (w/v) geraniol. Data are given in Supplementary Table S2.

## Data Availability Statement

The datasets presented in this study can be found in online repositories. The names of the repository/repositories and accession number(s) can be found below: https://www.ncbi.nlm.nih.gov/, SRR11285961 and https://www.ncbi.nlm.nih.gov/, SRR11285962.

## Author Contributions

JG, ET, NS, and GB conceived the study. JG, HF, DS, TH, AP, ZZ, ET, and GB designed the experiments. JG, HF, MW, AP, and ZZ performed the experiments. JG, HF, DS, ZZ, and ET analyzed the data. JG, HF, DS, AP, NS, and GB wrote the manuscript. All authors read and approved the final manuscript.

## Conflict of Interest

TH was employed by the company Thermo Fisher Scientific. The remaining authors declare that the research was conducted in the absence of any commercial or financial relationships that could be construed as a potential conflict of interest.
