# Supplementary material for: Production of the Fragrance Geraniol in Peroxisomes of a Product-Tolerant Baker’s Yeast
Source: Front Bioeng Biotechnol. 2020 Sep 23;8:582052. doi: 10.3389/fbioe.2020.582052 (PMC7546902; doi:10.3389/fbioe.2020.582052)
Supplement: Supplementary file 1 [file Presentation_1.pdf]

Supplementary Material

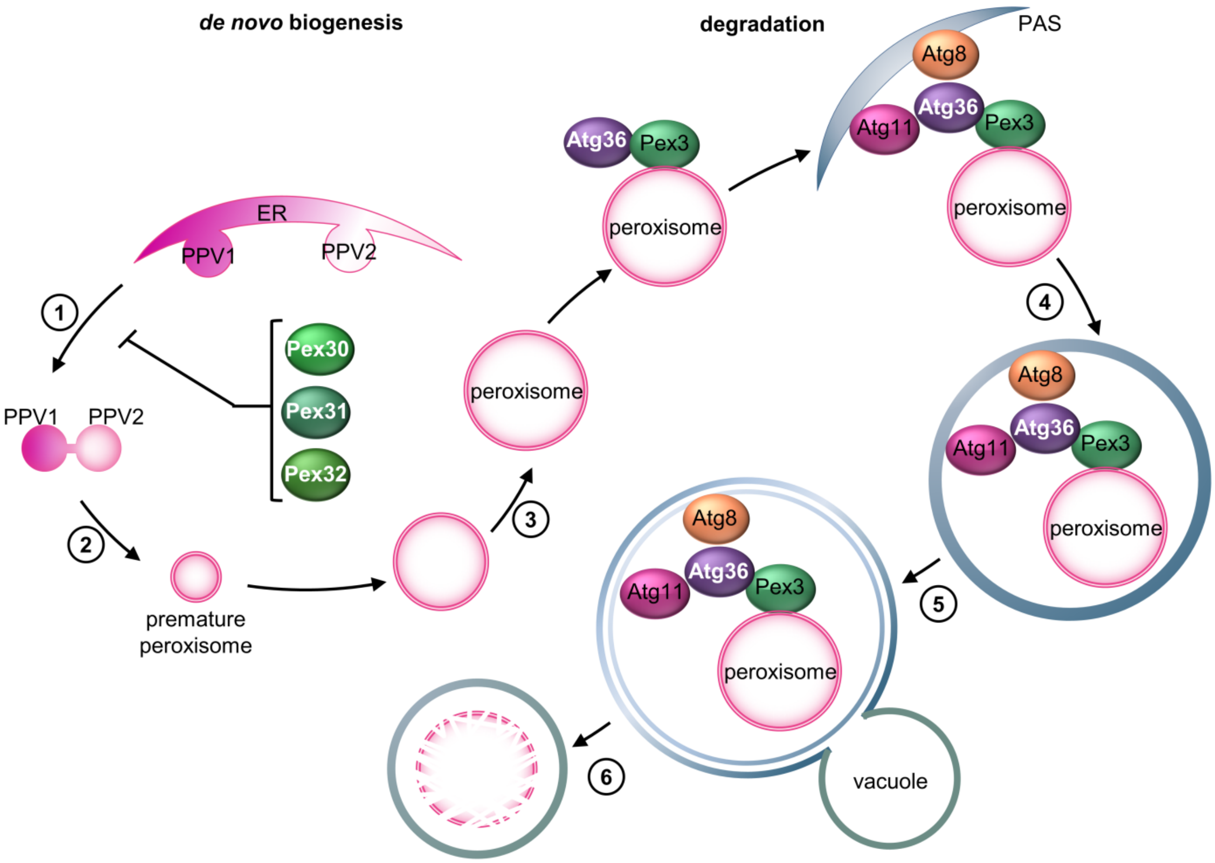

Supplementary Figure S1. Peroxisome *de novo* biogenesis from the endoplasmic reticulum (ER) and degradation. New peroxisomes are formed by constriction of two heterotypic pre-peroxisomal vesicles (PPV) from the ER (step 1). The two PPVs fuse to form a premature peroxisome (step 2), which matures by the import of matrix proteins (step 3). The number of peroxisomes is controlled by Pex30, Pex31, and Pex32. The peroxisome degradation, called pexophagy, is controlled by the specific pexophagy receptor Atg36. The peroxisomal membrane protein Pex3 recruits ATG36. The complex is targeted to the phagophore assembly site (PAS), where Atg36 interacts with Atg8 and Atg11 of the core autophagic machinery. At the PAS, a double membrane originates that engulfs the peroxisome (step 4), subsequently fuses with the vacuole (step 5) and finally the peroxisome is degraded (step 6). Adapted from (Yan et al., 2005; Motley et al., 2012; Agrawal and Subramani, 2013).

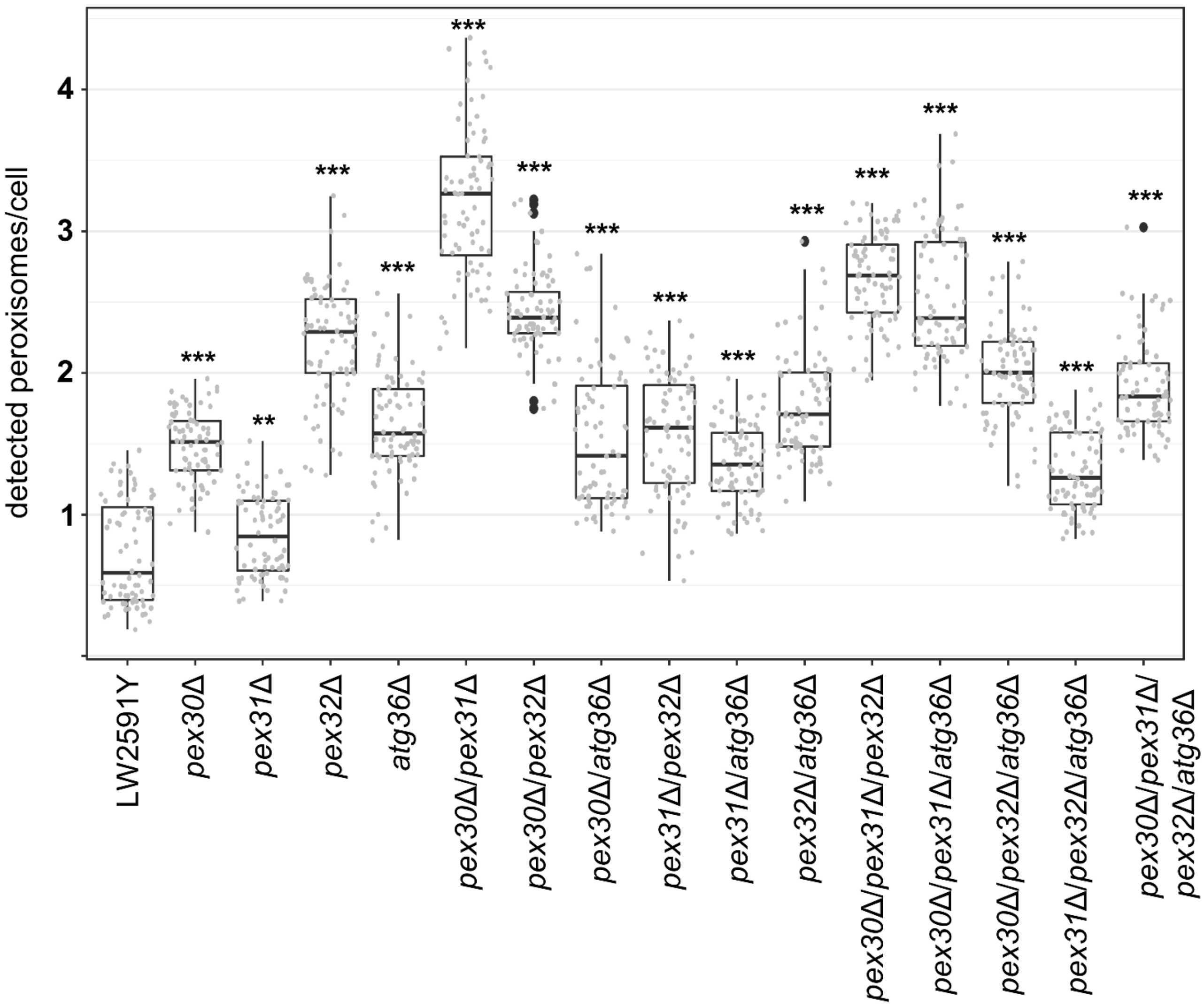

Supplementary Figure S2. Tukey's box whisker plot from Figure 2B with all 76 data points for the peroxisome/cell ratio per strain depicted. Peroxisomes per yeast cell were detected via fluorescence microscopy in the parental strain LW2591Y and all *PEX30*, *PEX31*, *PEX32*, and *ATG36* deletion combinations after introduction of a mCherry-SKL protein in YPD medium. Due to the reiterative recombination system site, all strains carry *gfp* integrated into the genome. For counting of the cells and peroxisomes in total 76 fluorescence images from two biological replicates were taken, the number of peroxisomes and cells were automatically counted, and the peroxisome/cell ratios were calculated for each image. The 76 ratios are depicted in the Tukey's box whisker plot. The p-value for each strain was calculated in comparison to LW2591Y (two-tailed two-sample T-test). *** = $p \leq 0.01$; ** = $p \leq 0.05$.

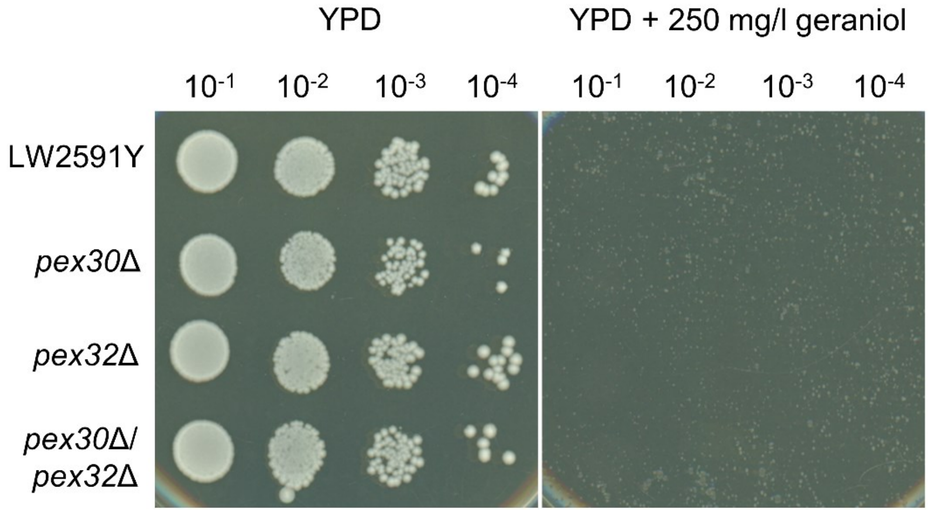

Supplementary Figure S3. The *S. cerevisiae* deletion strains *pex30*Δ, *pex32*Δ, and *pex30*Δ/*pex32*Δ are not growing on 250 mg/l geraniol. Spot-test of the reference strain LW2591Y and the deletion strains on YPD and on YPD with 250 mg/l geraniol. A serial dilution was used (OD = 10^{-1}, 10^{-2}, 10^{-3}, 10^{-4}). The plates were incubated for 3 days at 30°C.

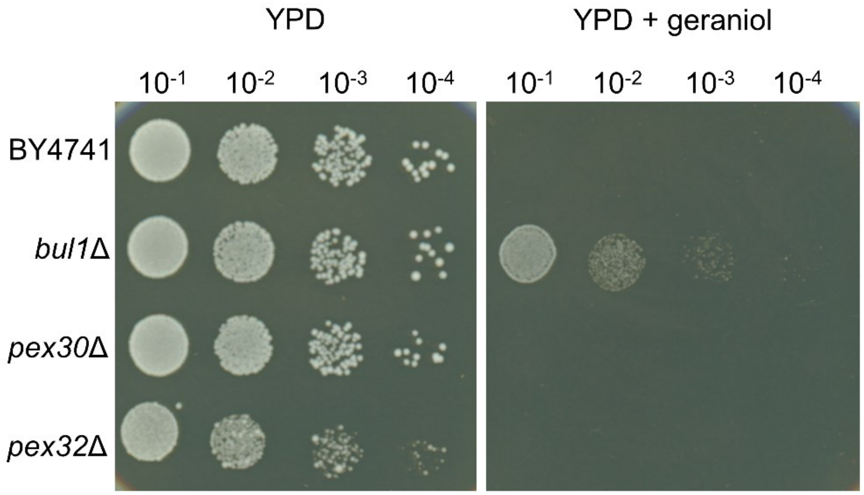

Supplementary Figure S4. The *S. cerevisiae BUL1* deletion strain is tolerant to geraniol. Spot-test of the reference strain BY4741 and the deletion strains *bul1*Δ, *pex30*Δ, and *pex32*Δ in the BY4741 background on YPD and on YPD with 200 mg/l geraniol. A serial dilution was used (OD = 10^{-1}, 10^{-2}, 10^{-3}, 10^{-4}). The plates were incubated for 3 days at 30°C.

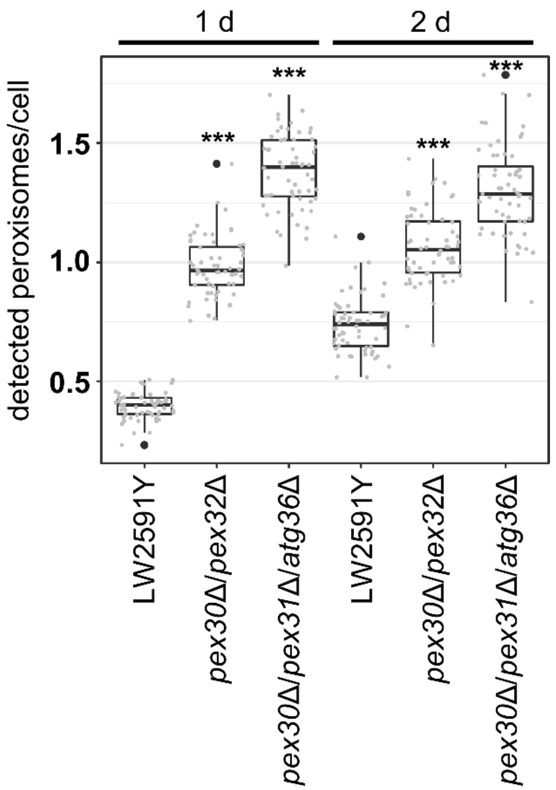

Supplementary Figure S5. Quantification of the number of peroxisomes per yeast cell in SC medium after 1 and 2 days, detected with fluorescence microscopy in the parental strain LW2591Y, *pex30*Δ/*pex32*Δ, and *pex30*Δ/*pex31*Δ/*atg36*Δ with integrated mCherry-SKL. For counting of the cells and peroxisomes, in total 60 fluorescence images from three biological replicates (20 images per replicate) were taken and the peroxisome/cell ratios were calculated. The 60 ratios are depicted in a Tukey's box whisker plot. The p-value for each strain was calculated in comparison to LW2591Y (two-tailed two-sample T-test). *** = $p \leq 0.01$.

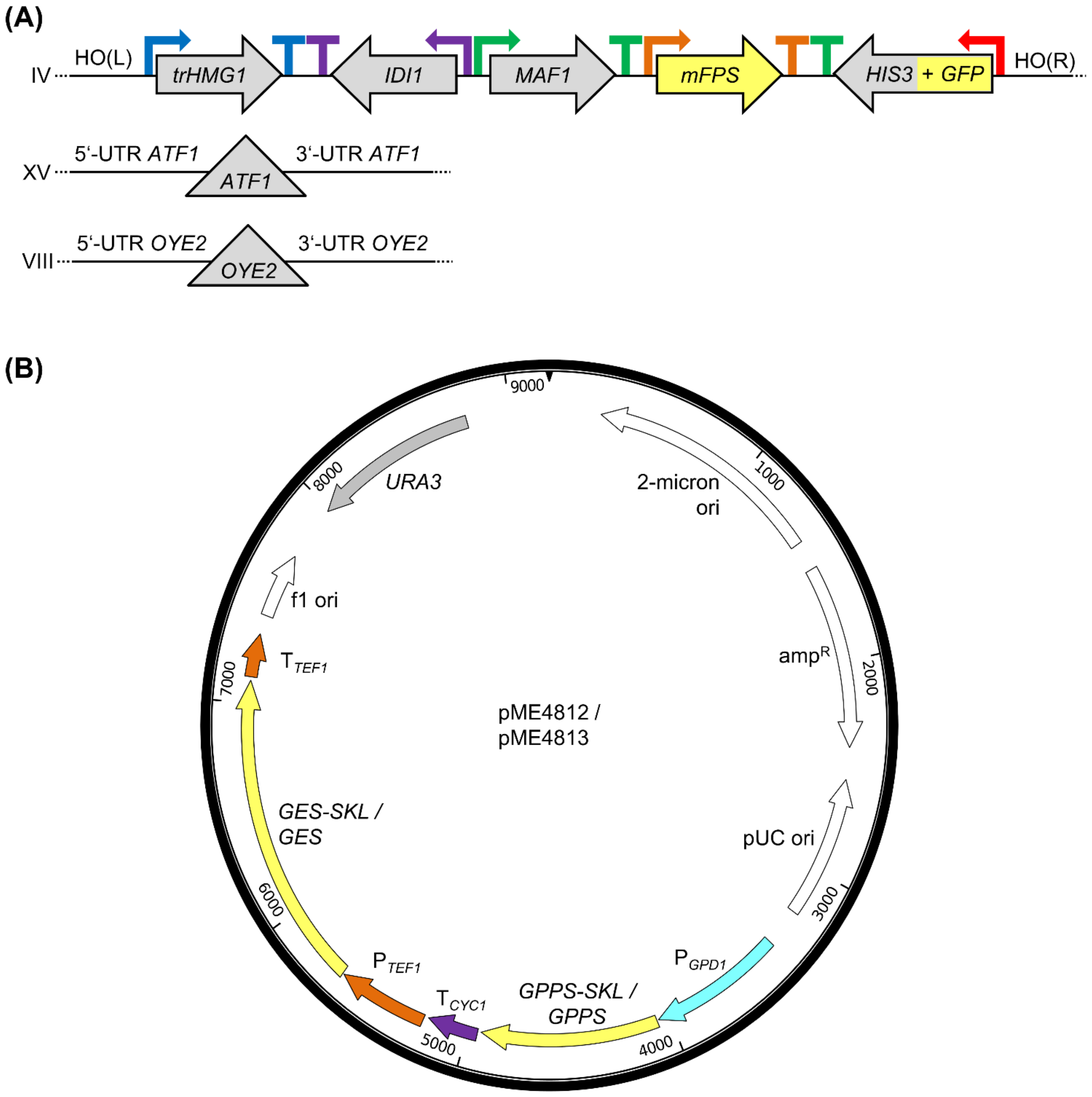

Supplementary Figure S6. Genome editing and plasmids inserted into the geraniol-tolerant and -sensitive *S. cerevisiae* strains. **(A)** Genes were integrated with the reiterative recombination method into the HO-locus on chromosome IV (Wingler and Cornish, 2011). *ATF1* and *OYE2* were deleted on chromosome XV and VIII, respectively. Colored bent arrows = promoters; colored T = terminators; linear grey arrows = *S. cerevisiae* sequences; linear yellow arrows = sequences from other organisms;

triangles = deleted genes. Dark blue = P_{URA3} and T_{URA3}, purple = P_{CYC1} and T_{CYC1}, green = P_{HIS3} and T_{HIS3}, orange = P_{TEF1} and T_{TEF1}, red = P_{PYK1}. **(B)** Map of high-copy plasmids pME4812 and pME4813 for peroxisomal and cytoplasmic localization of the GPP-synthase (GPPS) and the geraniol-synthase (GES). For peroxisomal localization in pME4812, the peroxisomal target sequence 1 (PTS1), consisting of the amino acids SKL was attached C-terminally to GPPS and GES. amp^R = ampicillin resistance gene, P = promoter, T = terminator, ori = origin of replication.

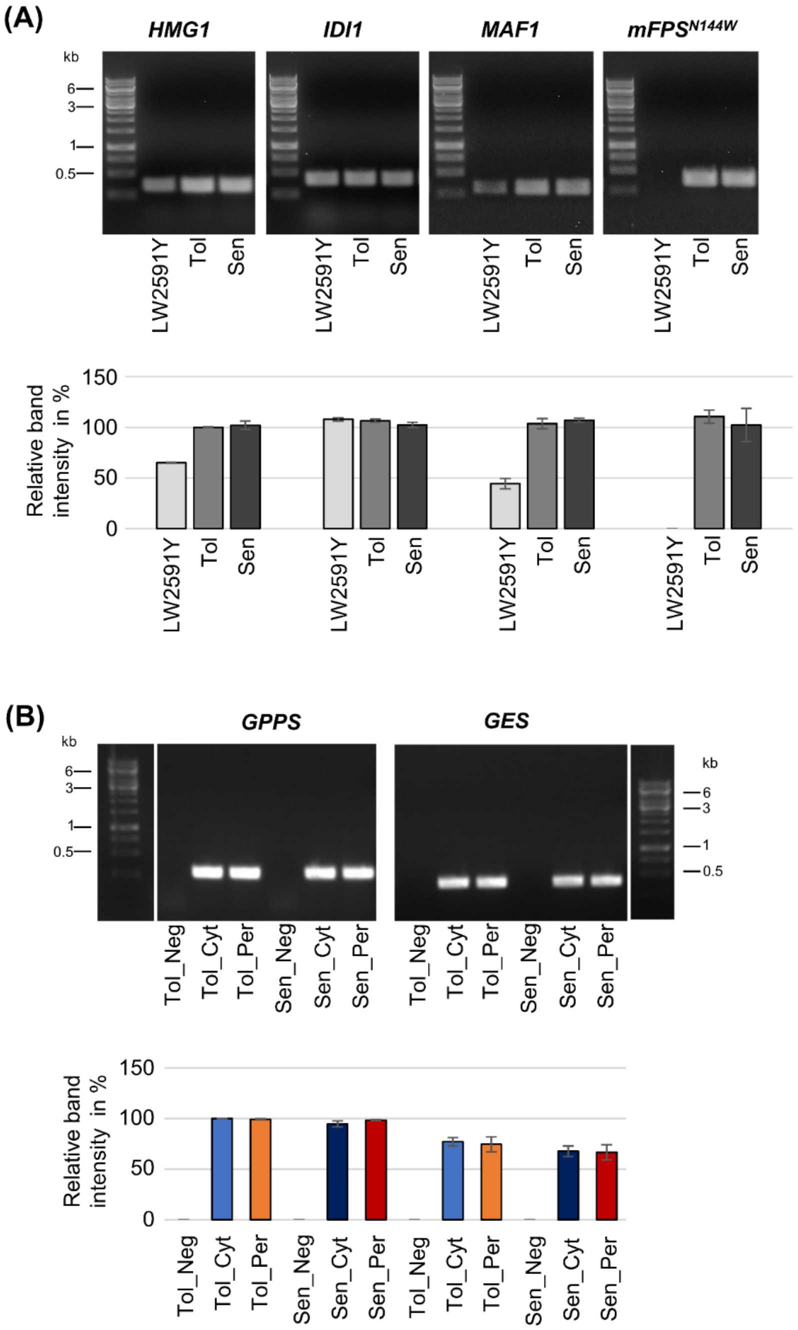

Supplementary Figure S7. Semiquantitative reverse transcription-PCR of **(A)** *HMG1*, *IDI1*, *MAF1*, and $mFPS^{N144W}$ in the strains LW2591Y, Tol, and Sen and **(B)** *GPPS* and *GES* in the strains Tol_Neg, Tol_Cyt, Tol_Per, Sen_Neg, Sen_Cyt, and Sen_Per. Semiquantitative reverse transcription-PCR was performed with cDNA of the indicated strains and with two biological replicates. The GeneRuler™ 1 kb DNA ladder (Thermo Fisher Scientific, SM0311) was used as size marker. The bands from two biological replicates were quantified with Fiji (Schindelin et al., 2012). The bands for *HMG1* in Tol and for *GPPS* in Tol_Cyt were set to 100%. The standard deviation is given.

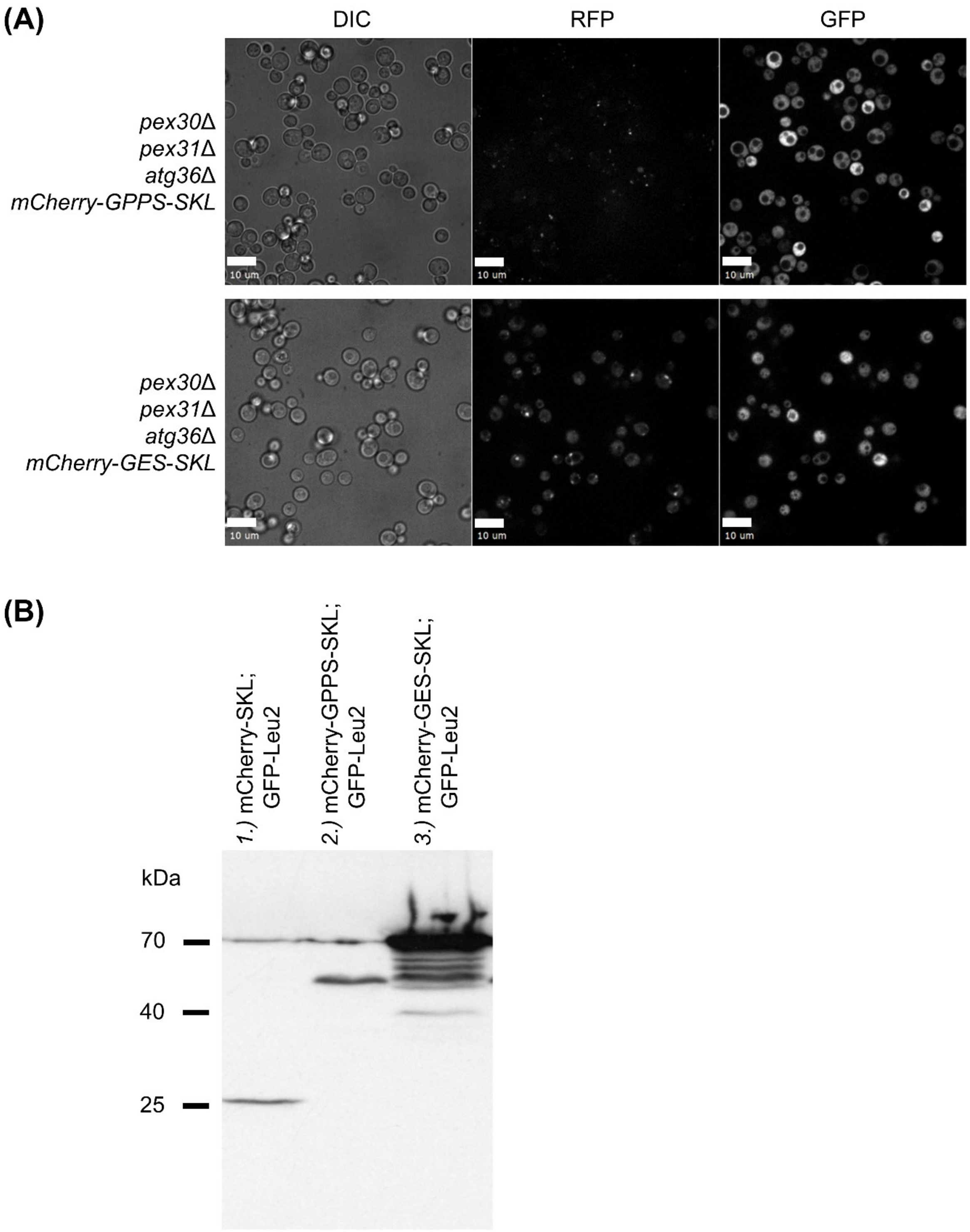

Supplementary Figure S8. Localization of mCherry-GPPS-SKL and mCherry-GES-SKL. **(A)** Fluorescence microscopy of *S. cerevisiae pex30*Δ/*pex31*Δ/*atg36*Δ with integrated *mCherry-GPPS-SKL* and *pex30*Δ/*pex31*Δ/*atg36*Δ with integrated *mCherry-GES-SKL*. Strains were grown 1 day in liquid SC

medium at 30°C, diluted and used for microscopy. Due to the reiterative recombination system site, all strains carry *gfp* integrated into the genome. Scale bar = 10 µm. **(B)** Western blot analysis of mCherry-GPPS-SKL and GES-SKL. α-RFP antibody was used, which shows a cross-reaction with GFP. All used strains carry a GFP fused to Leu2 due to the reiterative recombination system. Lane 1: Control strain RH3726 (carries mCherry-SKL (27 kDa) and GFP-Leu2 (67 kDa)), lane 2: *pex30*Δ/*pex31*Δ/*atg36*Δ/*mCherry-GPPS-SKL* (carries mCherry-GPPS-SKL (59 kDa) and GFP-Leu2), lane 3: *pex30*Δ/*pex31*Δ/*atg36*Δ/*mCherry-GES-SKL* (carries mCherry-GES-SKL (82 kDa) and GFP-Leu2).

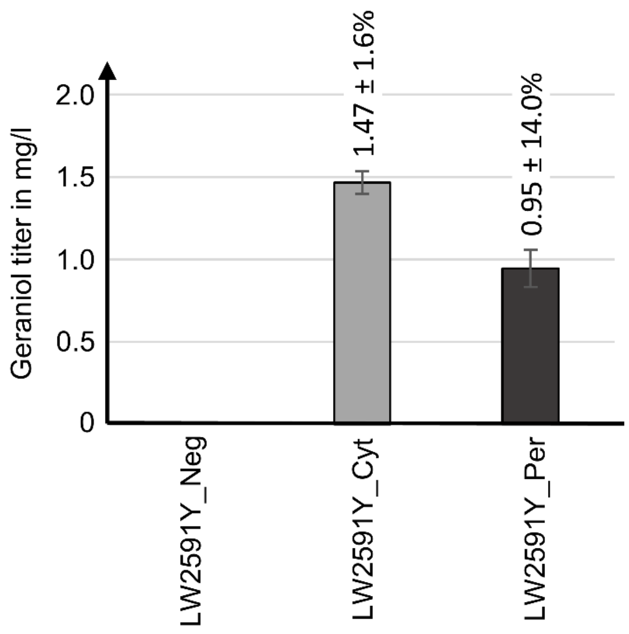

Supplementary Figure S9. Absolute geraniol titers of LW2591Y_Neg, LW2591Y_Cyt, and LW2591Y_Per. Data give the means with percentage differences from two biological replicates with each two technical GCMS replicates.

Supplementary Table S1. Strains constructed and used in this study.

**Name**	**Alternative name**	**Genotype**	**Reference**
LW2591Y		reiterative recombination parental acceptor strain; *MAT-a inc*; *his3Δ200*; *leu2Δ*; *met15Δ*; *trp1Δ63*; *ura3Δ*; P_{PYK}-*GFP-HIS3*-(HO cleavage site)	(Wingler and Cornish, 2011)
RH3711	*pex30Δ*	LW2591Y; *pex30Δ*; *BUL1*(G→T at 1579 bp)	this study
RH3712	*pex31Δ*	LW2591Y; *pex31Δ*	this study
RH3713	*pex32Δ*	LW2591Y; *pex32Δ*; *BUL1*(+A at 2125 bp)	this study
RH3714	*atg36Δ*	LW2591Y; *atg36Δ*	this study
RH3715	*pex30Δ/pex31Δ*	LW2591Y; *pex30Δ*; *pex31Δ*	this study
RH3716	*pex30Δ/pex32Δ*	LW2591Y; *pex30Δ*; *pex32Δ*; *BUL1*(+T at 2125 bp)	this study
RH3717	*pex30Δ/atg36Δ*	LW2591Y; *pex30Δ*; *atg36Δ*	this study
RH3718	*pex31Δ/pex32Δ*	LW2591Y; *pex31Δ*; *pex32Δ*	this study
RH3719	*pex31Δ/atg36Δ*	LW2591Y; *pex31Δ*; *atg36Δ*	this study
RH3720	*pex32Δ/atg36Δ*	LW2591Y; *pex32Δ*; *atg36Δ*	this study
RH3721	*pex30Δ/pex31Δ/ pex32Δ*	LW2591Y; *pex30Δ*; *pex31Δ*; *pex32Δ*	this study
RH3722	*pex30Δ/pex31Δ/ atg36Δ*	LW2591Y; *pex30Δ*; *pex31Δ*; *atg36Δ*	this study
RH3723	*pex30Δ/pex32Δ/ atg36Δ*	LW2591Y; *pex30Δ*; *pex32Δ*; *atg36Δ*	this study
RH3724	*pex31Δ/pex32Δ/ atg36Δ*	LW2591Y; *pex31Δ*; *pex32Δ*; *atg36Δ*	this study
RH3725	*pex30Δ/pex31Δ/ pex32Δ/atg36Δ*	LW2591Y; *pex30Δ*; *pex31Δ*; *pex32Δ*; *atg36Δ*	this study
RH3726	LW2591Y/ *mCherry-SKL*	LW2591Y; P_{GPD1}-*mCherry-SKL*-T_{CYC1}; *GFP-LEU2*	this study
RH3727	*pex30Δ/mCherry -SKL*	LW2591Y; *pex30Δ*; P_{GPD1}-*mCherry-SKL*-T_{CYC1}; *GFP-LEU2*; *BUL1*(G→T at 1579 bp)	this study
RH3728	*pex31Δ/mCherry -SKL*	LW2591Y; *pex31Δ*; P_{GPD1}-*mCherry-SKL*-T_{CYC1}; *GFP-LEU2*	this study
RH3729	*pex32Δ/mCherry -SKL*	LW2591Y; *pex32Δ*; P_{GPD1}-*mCherry-SKL*-T_{CYC1}; *GFP-LEU2*; *BUL1*(+A at 2125 bp)	this study
RH3730	*atg36Δ/mCherry -SKL*	LW2591Y; *atg36Δ*; P_{GPD1}-*mCherry-SKL*-T_{CYC1}; *GFP-LEU2*	this study
RH3731	*pex30Δ/pex31Δ/ mCherry-SKL*	LW2591Y; *pex30Δ*; *pex31Δ*; P_{GPD1}-*mCherry-SKL*-T_{CYC1}; *GFP-LEU2*	this study
RH3732	*pex30Δ/pex32Δ/ mCherry-SKL*	LW2591Y; *pex30Δ*; *pex32Δ*; P_{GPD1}-*mCherry-SKL*-T_{CYC1}; *GFP-LEU2*; *BUL1*(+T at 2125 bp)	this study
RH3733	*pex30Δ/atg36Δ/ mCherry-SKL*	LW2591Y; *pex30Δ*; *atg36Δ*; P_{GPD1}-*mCherry-SKL*-T_{CYC1}; *GFP-LEU2*	this study

Name	Alternative name	Genotype	Reference
RH3734	*pex31Δ/pex32Δ/ mCherry-SKL*	LW2591Y; *pex31Δ*; *pex32Δ*; P_{GPD1}-*mCherry-SKL*-T_{CYC1}; *GFP-LEU2*	this study
RH3735	*pex31Δ/atg36Δ/ mCherry-SKL*	LW2591Y; *pex31Δ*; *atg36Δ*; P_{GPD1}-*mCherry-SKL*-T_{CYC1}; *GFP-LEU2*	this study
RH3736	*pex32Δ/atg36Δ/ mCherry-SKL*	LW2591Y; *pex32Δ*; *atg36Δ*; P_{GPD1}-*mCherry-SKL*-T_{CYC1}; *GFP-LEU2*	this study
RH3737	*pex30Δ/pex31Δ/ pex32Δ/ mCherry-SKL*	LW2591Y; *pex30Δ*; *pex31Δ*; *pex32Δ*; P_{GPD1}-*mCherry-SKL*-T_{CYC1}; *GFP-LEU2*	this study
RH3738	*pex30Δ/pex31Δ/ atg36Δ/ mCherry-SKL*	LW2591Y; *pex30Δ*; *pex31Δ*; *atg36Δ*; P_{GPD1}-*mCherry-SKL*-T_{CYC1}; *GFP-LEU2*	this study
RH3739	*pex30Δ/pex32Δ/ atg36Δ/ mCherry-SKL*	LW2591Y; *pex30Δ*; *pex32Δ*; *atg36Δ*; P_{GPD1}-*mCherry-SKL*-T_{CYC1}; *GFP-LEU2*	this study
RH3740	*pex31Δ/pex32Δ/ atg36Δ/ mCherry-SKL*	LW2591Y; *pex31Δ*; *pex32Δ*; *atg36Δ*; P_{GPD1}-*mCherry-SKL*-T_{CYC1}; *GFP-LEU2*	this study
RH3741	*pex30Δ/pex31Δ/ pex32Δ/atg36Δ/ mCherry-SKL*	LW2591Y; *pex30Δ*; *pex31Δ*; *pex32Δ*; *atg36Δ*; P_{GPD1}-*mCherry-SKL*-T_{CYC1}; *GFP-LEU2*	this study
RH3743	Tol	LW2591Y; *pex30Δ*; *pex32Δ*; P_{URA3}-*trHMG1*-T_{URA3}; P_{CYC1}-*IDI1*-T_{CYC1}; P_{HIS3}-*MAF1*-T_{HIS3}; P_{TEF1}-*mFPS*^{N144W}-T_{TEF1}; *atf1Δ*; *oye2Δ*; *GFP-HIS3*; *BUL1*(+T at 2125 bp)	this study
RH3744	Sen	LW2591Y; *pex30Δ*; *pex31Δ*; *atg36Δ*; P_{URA3}-*trHMG1*-T_{URA3}; P_{CYC1}-*IDI1*-T_{CYC1}; P_{HIS3}-*MAF1*-T_{HIS3}; P_{TEF1}-*mFPS*^{N144W}-T_{TEF1}; *atf1Δ*; *oye2Δ*; *GFP-HIS3*	this study
RH3745	LW2591_Neg	LW2591Y; P_{URA3}-*trHMG1*-T_{URA3}; P_{CYC1}-*IDI1*-T_{CYC1}; P_{HIS3}-*MAF1*-T_{HIS3}; P_{TEF1}-*mFPS*^{N144W}-T_{TEF1}; pME4814; *GFP-HIS3*	this study
RH3746	LW2591_Cyt	LW2591Y; P_{URA3}-*trHMG1*-T_{URA3}; P_{CYC1}-*IDI1*-T_{CYC1}; P_{HIS3}-*MAF1*-T_{HIS3}; P_{TEF1}-*mFPS*^{N144W}-T_{TEF1}; pME4813; *GFP-HIS3*	this study
RH3747	LW2591_Per	LW2591Y; P_{URA3}-*trHMG1*-T_{URA3}; P_{CYC1}-*IDI1*-T_{CYC1}; P_{HIS3}-*MAF1*-T_{HIS3}; P_{TEF1}-*mFPS*^{N144W}-T_{TEF1}; pME4812; *GFP-HIS3*	this study
RH3748	Tol_Neg	LW2591Y; *pex30Δ*; *pex32Δ*; P_{URA3}-*trHMG1*-T_{URA3}; P_{CYC1}-*IDI1*-T_{CYC1}; P_{HIS3}-*MAF1*-T_{HIS3}; P_{TEF1}-*mFPS*^{N144W}-T_{TEF1}; *atf1Δ*; *oye2Δ*; pME4814; *GFP-HIS3*; *BUL1*(+T at 2125 bp)	this study
RH3749	Tol_Cyt	LW2591Y; *pex30Δ*; *pex32Δ*; P_{URA3}-*trHMG1*-T_{URA3}; P_{CYC1}-*IDI1*-T_{CYC1}; P_{HIS3}-*MAF1*-T_{HIS3}; P_{TEF1}-*mFPS*^{N144W}-T_{TEF1}; *atf1Δ*; *oye2Δ*; pME4813; *GFP-HIS3*; *BUL1*(+T at 2125 bp)	this study
RH3750	Tol_Per	LW2591Y; *pex30Δ*; *pex32Δ*; P_{URA3}-*trHMG1*-T_{URA3}; P_{CYC1}-*IDI1*-T_{CYC1}; P_{HIS3}-*MAF1*-T_{HIS3}; P_{TEF1}-*mFPS*^{N144W}-T_{TEF1}; *atf1Δ*; *oye2Δ*; pME4812; *GFP-HIS3*; *BUL1*(+T at 2125 bp)	this study
RH3751	Sen_Neg	LW2591Y; *pex30Δ*; *pex31Δ*; *atg36Δ*; P_{URA3}-*trHMG1*-T_{URA3}; P_{CYC1}-*IDI1*-T_{CYC1}; P_{HIS3}-*MAF1*-T_{HIS3}; P_{TEF1}-*mFPS*^{N144W}-T_{TEF1}; *atf1Δ*; *oye2Δ*; pME4814; *GFP-HIS3*	this study

**Name**	**Alternative name**	**Genotype**	**Reference**
RH3752	Sen_Cyt	LW2591Y; *pex30*Δ; *pex31*Δ; *atg36*Δ; P_{URA3}-*trHMG1*-T_{URA3}; P_{CYC1}-*IDI1*-T_{CYC1}; P_{HIS3}-*MAF1*-T_{HIS3}; P_{TEF1}-*mFPS*^{N144W}-T_{TEF1}; *atf1*Δ; *oye2*Δ; pME4813; *GFP-HIS3*	this study
RH3753	Sen_Per	LW2591Y; *pex30*Δ; *pex31*Δ; *atg36*Δ; P_{URA3}-*trHMG1*-T_{URA3}; P_{CYC1}-*IDI1*-T_{CYC1}; P_{HIS3}-*MAF1*-T_{HIS3}; P_{TEF1}-*mFPS*^{N144W}-T_{TEF1}; *atf1*Δ; *oye2*Δ; pME4812; *GFP-HIS3*	this study
RH3802	*pex30*Δ/*pex31*Δ/ *atg36*Δ/ *mCherry-GPPS-SKL*	LW2591Y; *pex30*Δ; *pex31*Δ; *atg36*Δ; P_{GPD1}-*mCherry-GPPS-SKL*-T_{CYC1}; *GFP-LEU2*	this study
RH3803	*pex30*Δ/*pex31*Δ/ *atg36*Δ/ *mCherry-GES-SKL*	LW2591Y; *pex30*Δ; *pex31*Δ; *atg36*Δ; P_{TEF1}-*mCherry-GES-SKL*-T_{TEF1}; *GFP-LEU2*	this study
BY4741		*MATa*; *his3*Δ*1*; *leu2*Δ*0*; *met15*Δ*0*; *ura3*Δ*0*	EURO-SCARF
Y00861	*bul1*Δ	BY4741; *MATa*; *his3*Δ*1*; *leu2*Δ*0*; *met15*Δ*0*; *ura3*Δ*0*; *YMR275c*::*kanMX4*	EURO-SCARF
Y05233	*pex30*Δ	BY4741; *MATa*; *his3*Δ*1*; *leu2*Δ*0*; *met15*Δ*0*; *ura3*Δ*0*; *YLR324w*::*kanMX4*	EURO-SCARF
Y07166	*pex32*Δ	BY4741; *MATa*; *his3*Δ*1*; *leu2*Δ*0*; *met15*Δ*0*; *ura3*Δ*0*; *YBR168w*::*kanMX4*	EURO-SCARF

Supplementary Table S2. Geraniol titer determination with GCMS. 0.002% (w/v) menthol were added as standard to the extracted geraniol samples. The peak areas of the menthol and the geraniol peaks were determined. The geraniol titer was calculated using the formula y = 0.0011*x + 0.00003, which was determined from the calibration line by measuring different concentrations of geraniol with 0.002% menthol.

**Strain**	**Peak area (Geraniol)**	**Peak area (Menthol)**	**Peak area (Geraniol)/ Peak area (Menthol)**	**Geraniol titer (%)**	**Geraniol titer (mg/l)**
Sen_Cyt	685985	5390517	0.127	0.0001700	1.700
	581497	5647801	0.103	0.0001433	1.433
	585938	5507083	0.106	0.0001470	1.470
	612289	5547277	0.110	0.0001514	1.514
Sen_Per	803488	5964075	0.135	0.0001782	1.782
	743674	5741172	0.130	0.0001725	1.725
	594142	5181605	0.115	0.0001561	1.561
	637011	5135492	0.124	0.0001664	1.664
Tol_Cyt	1126052	5452509	0.207	0.0002572	2.572
	1017663	5576591	0.182	0.0002307	2.307
	1097505	5549117	0.198	0.0002476	2.476
	1102622	5772022	0.191	0.0002401	2.401
Tol_Per	1354407	6031957	0.225	0.0002770	2.770
	1281521	5721703	0.224	0.0002764	2.764
	1319961	5676361	0.233	0.0002858	2.858
	1183663	5650041	0.209	0.0002604	2.604
LW2591Y_Cyt	597123	5561374	0.107	0.0001481	1.481
	605593	5913414	0.102	0.0001427	1.427
	640668	5555178	0.115	0.0001569	1.569
	531117	5379496	0.099	0.0001386	1.386
LW2591Y_Per	449495	5919915	0.076	0.0001135	1.135
	319957	5986446	0.053	0.0000888	0.888
	283562	5776123	0.049	0.0000840	0.840
	317737	5649604	0.056	0.0000919	0.919

Supplementary Table S3. Plasmids constructed and used in this study.

Name	Alternative name	Description	Reference
pBluescript II KS (+)		Cloning vector, *amp*^{R}	Thermo Fisher Scientific
pESC_URA		Cloning vector	Stratagene
pME4803	pJG105	P_{GPD1}-*mCherry*-*SKL*-T_{CYC1}	this study
pME4804	pJG110	P_{TEF1}-*mFPS*^{N144W}-T_{TEF1}	this study
pME4805	pJG111	P_{HIS3}-*GES*-*SKL*-T_{HIS3}	this study
pME4806	pJG112	P_{CYC1}-*GPPS*-*SKL*-T_{CYC1}	this study
pME4807	pJG115	P_{URA3}-*trHMG1*-T_{URA3}	this study
pME4808	pJG116	P_{CYC1}-*IDI1*-T_{CYC1}	this study
pME4809	pJG117	P_{HIS3}-*MAF1*-T_{HIS3}	this study
pME4810	pJG178	P_{GPD1}-*GPPS*-*SKL*-T_{CYC1}	this study
pME4811	pJG180	P_{GPD1}-*GPPS*- T_{CYC1}	this study
pME4812	pJG199, Per	P_{GPD1}-*GPPS*-*SKL*- T_{CYC1}; P_{TEF1}-*GES*-*SKL*-T_{TEF1}	this study
pME4813	pJG200, Cyt	P_{GPD1}-*GPPS*-T_{CYC1}; P_{TEF1}-*GES*-T_{TEF1}	this study
pME4814	pJG223, Neg	pESC (*Pvu*II), religated	this study
pME5088	pJG132	P_{GPD1}-*mCherry*-*GPPS*-*SKL*-T_{CYC1}	this study
pME5089	pJG133	P_{TEF1}-*mCherry*-*GES*-*SKL*-T_{TEF1}	this study
pME2787		carries *CYC1* promoter and terminator and *URA3* marker	(Lin et al., 2015)
pLW2592		odd donor plasmid for reiterative recombination	(Wingler and Cornish, 2011)
pLW2593		even donor plasmid for reiterative recombination	(Wingler and Cornish, 2011)
pLW2594		round 1 donor plasmid for reiterative recombination	(Wingler and Cornish, 2011)
pME4093		carries *GPD1* promoter	(Shahpasandzadeh et al., 2014)
pME3772		carries *mCherry* gene	(Petroi et al., 2012)

Supplementary Table S4. Primers used in this study.

**Primer name**	**Sequence (5' → 3')**	**Nucleotide number**
JG435	TCA GTA CAA TCT TAG GGA TAA CAG GGT AAT	30
JG436	TGA GAA GGT TTT GGG ACG CTC GAA GGC TTT	30
JG437	GCA CAG TTA TAC TGT TGC GGA AAG CTG AAA	30
JG933	ATG AAA TTG TTA TGG AGC TTA GTG	24
JG934	ACT ATC AAA TAA AGG GAA ACC AAG	24
JG935	CTT GGT TTC CCT TTA TTT GAT AGT TGA GTG CAT GCC CCA TAT GGA CAA CAA CGT TCA CAC TGG TTT CAA TTC ATC ATT TTT TTT TT	86
JG936	TTA TAG AGA AGG AGA CAT TGA AAC ATC AGA TAT TTG TAT TCC TGA TGC GGT ATT TTC	57
JG937	TTA TAG AGA AGG AGA CAT TGA AAC	24
JG938	TAC TTC ATC AAG AAC TTC TAT AGT	24
JG939	TTC TTT CCT TGA AAT TAG CAA AAT	24
JG940	ATT TTG CTA ATT TCA AGG AAA GAA TAG TAT TGC TTT AAG CCT CTA AAC TGC ATA TAT AGT TGG TTT CAA TTC ATC ATT TTT TTT TT	86
JG941	TTA CAA ATG GAA GAG GCG TCG TTT CCA TTT TCT TGA CCT GCC TGA TGC GGT ATT TTC	57
JG942	TTA CAA ATG GAA GAG GCG TCG	21
JG943	TTG GTT CAG CTT CCA GCC AGT C	22
JG944	TGA AAA TCA GTT TTT ACA CTC CGG	24
JG945	CCG GAG TGT AAA AAC TGA TTT TCA TGC TCG CTC CCG TTT TTA CCT TTA CAT AAT ATA ATC TCT ATT CAA TTC ATC ATT TTT TTT TT	86
JG946	TCA TAC GGC CTT CTT GCT ATC GCG ACC AAT GGT TGG ATT TCC TGA TGC GGT ATT TTC	57
JG947	TCA TAC GGC CTT CTT GCT ATC G	22
JG965	ATC GAT AAG CTT GAT AGT TTA TCA TTA TCA ATA CTC GCC A	40
JG969	CTG CAG GAA TTC GAT GCA AAT TAA AGC CTT CGA GCG T	37
JG972	AAA ATT GTG CCT TTG GAC TTA AAA TGG CGT AGT TTA TCA TTA TCA ATA CTC GCC A	55
JG973	CTT AGG GAT AAC AGG GTA ATG CAA ATT AAA GCC TTC GAG CGT	42
JG986	CCT GGA TCC GCT TCT CGT CTG	21
JG987	TAA TGA GCT GTA CTG ACC ACC AAA	24
JG994	TTT GGT GGT CAG TAC AGC TCA TTA AAG AAA GTT AGA TAC CCT CAT TCT AAA ACG TTG TCC GAA CTT CAA TTC ATC ATT TTT TTT TT	86
JG995	CTA CCA TTC TCT GTC CAA AAA CCT AGA AAG TAA AGT CCA GCC TGA TGC GGT ATT TTC	57
JG996	CTA CCA TTC TCT GTC CAA AAA CCT	24
JG1062	GCC CTT GCT CAC CAT ATC CGT CGA AAC TAA GTT CTG G	37
JG1063	ATG GTG AGC AAG GGC GAG GA	20
JG1064	CTA CAA CTT GGA CTT GTA CAG CTC GTC CAT GC	32
JG1065	AAG TCC AAG TTG TAG TCA TGT AAT TAG TTA TGT CAC GCT	39

Primer name	Sequence (5' → 3')	Nucleotide number
JG1137	TCA TGT AAT TAG TTA TGT CAC GCT	24
JG1172	CAT ATC CGT CGA AAC TAA GTT CT	23
JG1183	GAA CTC TTA AAG CGA TTT ATG AGA	24
JG1184	TAT CGA TAC TAT ATT ATC GTC TAT AT	26
JG1185	ATA TAG ACG ATA ATA TAG TAT CGA TAT AGT GTT AAC CGT ACT TTG TAG CAC CAT TTC TTT TTC TTC TTC AAT TCA TCA TTT TTT TTT T	88
JG1186	TTA ATT TTT GTC CCA ACC GAG TTT TAG AGC TTC TTC GTA CCC TGA TGC GGT ATT TTC	57
JG1187	TTA ATT TTT GTC CCA ACC GAG TTT T	25
JG1188	GAC AGT GAA CTA CAG GAA TAT AC	23
JG1189	GAG AGC TGA TAA ATT GAT GGT ATT T	25
JG1190	AAA TAC CAT CAA TTT ATC AGC TCT CAT CTC ACA TGA TGC TTG ACT GAT ATT ATT CGA CAA TAT GAT TCA ATT CAT CAT TTT TTT TTT	87
JG1191	CTA AGG GCC TAA AAG GAG AGC TTT GTA AAT GGA GCA AAG CCC TGA TGC GGT ATT TTC	57
JG1192	CTA AGG GCC TAA AAG GAG AGC	21
JG1220	GAA CTT AGT TTC GAC GGA TAT GGA ATT CGA CTT CAA CAA GTA C	43
JG1221	GAC ATA ACT AAT TAC ATG ATT ACA ACT TAG AGT TTT GTC TGA AA	44
JG1224	AAT ACA CAC ACT AAT CGT TTA TGA CTG CCG ACA ACA ATA GTA T	43
JG1225	ACA TAA CTA ATT ACA TGT TTT TAT AGC ATT CTA TGA ATT TGC CTG	45
JG1226	ATA AAC GAA GGC AAA GGT TTA TGA AAG TAT GTT ATC ACT CTA AAA C	46
JG1227	TAA ATA ATC GGT GTC AGT TTC TAC TGT AGG GAT TCT TCT TGA T	43
JG1228	ACG GTA TCG ATA AGC TTG ATT TCA ATT CAT CAT TTT TTT TTT ATT CTT	48
JG1229	CCG GGC TGC AGG AAT TCG ATC CTG ATG CGG TAT TTT CTC CTT	42
JG1230	TTT CAC CAA TTG GTC CAT GTT TAA ACG ATT TAT CTT CGT TTC CTG CAG G	49
JG1231	TGC ATT AAA TCC TAA GTT TAA ACA AAA CTG TAT TAT AAG TAA ATG CAT G	49
JG1232	GAC CAA TTG GTG AAA ACT GAA GT	23
JG1233	TTA GGA TTT AAT GCA GGT GAC GG	23
JG1234	GAC ATA ACT AAT TAC ATG ATT AGT TTT GTC TGA AAG CAA CAT AAT CA	47
JG1259	GGA CGC TCG AAG GCT TTC CAA CCA ACG CTC GCC AAA TTA AAT TCC CGT TTT AAG AGC TTG G	61
JG1262	CTG TTG CGG AAA GCT GAA AAG TAT TGA TAA TGA TAA ACT CAG TAT AGC GAC CAG CAT TCA C	61
JG1263	AAA ATT GTG CCT TTG GAC TTA AAA TGG CGT TTC AAT TCA TCA TTT TTT TTT TAT TCT T	58
JG1269	ATG GAA GAA TCC TCC AGT AAG AG	23
JG1301	CTC TTT CTG ATT GCG TCC TTT T	22
JG1304	CAC GAA AAC GCT AAG GCT ATC T	22

Primer name	Sequence (5' → 3')	Nucleotide number
JG1318	GTT TTG TCT GAA AGC AAC ATA ATC A	25
JG1320	TTG GGT GAA GAA CAA AGC ATC AA	23
JG1329	CTT AGG GAT AAC AGG GTA ATG CTC GAA GGC TTT AAT TTG CCC TGA TGC GGT ATT TTC TCC TT	62
JG1343	GGA CGC TCG AAG GCT TTG GAG AAA ATA CCG CAT CAG GGC AAA TTA AAG CCT TCG AGC GT	59
JG1344	CTG TTG CGG AAA GCT GAA AGC TCT TAA AAC GGG AAT TTA ATT TGG CGA GCG TTG GTT GGT G	61
JG1347	CTT AGG GAT AAC AGG GTA ATT TGA AGC TAT GGT GTG TGG GTA TGA TCC GTC GAG TTC AAG AGA	63
JG1348	GGA CGC TCG AAG GCT TTC TTG AAC TCG ACG GAT CAT ACC CAC ACA CCA TAG CTT CAA AAT	60
JG1355	TGG CCG ATT CAT TAA TGC AGA GTT TAT CAT TAT CAA TAC TCG CCA	45
JG1357	TAT TCA CCC AAA AAC CAT ACC C	22
JG1384	GAA GGC TTT AAT TTG CGT TTC CCA CAC ACC ATA GCT TCA AAA T	43
JG1385	TTA CTG GAG GAT TCT TCC ATA AAC TTA GAT TAG ATT GCT ATG CTT T	46
JG1386	TCA TTG GGT GAA GAA CAA AGC AT	23
JG1388	CTT CGC TAT TAC GCC AGT TTC AGT ATA GCG ACC AGC ATT CAC	42
JG1389	TCA CAA CTT GGA TTG GGT GAA GA	23
JG1390	TCA CCC AAT CCA AGT TGT GAT CAG TAC TGA CAA TAA AAA GAT TCT T	46
JG1515	GTG GTG CTA TTA TTG GTG GTG C	22
JG1656	TTA TAG CAT TCT ATG AAT TTG CCT G	25
JG1658	CTA CTG TAG GGA TTC TTC TTG AT	23
JG1659	GAA GGG TAA GCT AGA CGA TAA G	22
JG1660	GAA GTG GAC GGT GAT TTG AGA A	22
JG1720	TCA CTT TTG TCT CTT GTA GAT CTT	24
JG1811	CTT GTA CAG CTC GTC CAT GCC	21
JG1874	GGA CCC CAA GCT CTG GAA GTA T	22
JG1882	ACT AGT GGA TCC CCC ATG GCC AAA GAT TTG AAC GAT TC	38
JG1883	GAA TTC CTG CAG CCC TTA TTT TGT CAC TTG CCT AAC AGA	39
JG1885	TCA GAC TCC CAA GAA ATT TTG CA	23
JG1910	AAT CTA AGT TTG TTT ATG GTG AGC AAG GGC GAG GA	35
JG1911	GAC GAG CTG TAC AAG GAA GAA TCC TCC AGT AAG AGA AG	38
JG1912	GTC AGT ACT GAG TTT TCA CAA CTT GGA TTG GGT GAA GA	38
JG1913	GAC GAG CTG TAC AAG GAA TTC GAC TTC AAC AAG TAC ATG	39
JG1961	AAA ATT GTG CCT TTG GAC TTA AAA TGG CGT CCC ACA CAC CAT AGC TTC AAA AT	53
JG1962	CTT AGG GAT AAC AGG GTA ATC AGT ATA GCG ACC AGC ATT CAC	42

Supplementary Table S5. Primers used for seamless gene deletion.

**Gene of interest**	**Forward and reverse primer for amplification of 5'-region**	**Forward and reverse primer for amplification of URA3-marker**	**Forward and reverse primer for fusion-PCR**
PEX30 deletion	JG943/944	JG945/946	JG943/947
PEX31 deletion	JG933/934	JG935/936	JG933/937
PEX32 deletion	JG938/939	JG940/941	JG938/942
ATG36 deletion	JG986/987	JG994/995	JG986/996
ATF1 deletion	JG1188/1189	JG1190/1191	JG1188/1192
OYE2 deletion	JG1183/1184	JG1185/1186	JG1183/1187

Supplementary Table S6. PCR parameters for the reiterative recombination system.

**Fragment for amplification**	**Primers for PCR 1**	**Template DNA for PCR 1**	**Primers for PCR 2**	**Linearized plasmid for transformation**
P_{GPD1}-*mCherry-SKL*-T_{CYC1}	JG972/973	pME4803	JG972/435	pLW2594 (*Xma*I)
P_{URA3}-*trHMG1*-T_{URA3}	JG1263/1329	pME4807	JG1263/435	pLW2594 (*Xma*I)
P_{CYC1}-*IDI1*-T_{CYC1}	JG1343/1344	pME4808	JG436/437	pLW2593 (*Xma*I)
P_{HIS3}-*MAF1*-T_{HIS3}	JG1259/1347	pME4809	JG436/435	pLW2592 (*Sac*I)
P_{TEF1}-*mFPS*^{N144W}-T_{TEF1}	JG1348/1262	pME4804	JG436/437	pLW2593 (*Xma*I)
P_{GPD1}-*mCherry-GPPS-SKL*-T_{CYC1}	JG972/973	pME5088	JG972/435	pLW2594 (*Xma*I)
P_{TEF1}-*mCherry-GES-SKL*-T_{TEF1}	JG1961/1962	pME5089	JG1961/435	pLW2594 (*Xma*I)

Sequences of synthesized genes, promoters and terminators:

>*mFPS*^{N144W}

ATGCACAAGTTCACCGGTGTTAACGCTAAATTTCAACAACCAGCTTTGAGAAACTTGTCC
CCAGTTGTTGTCGAAAGAGAAAGAGAAGAATTCGTTGGATTCTTCCCACAAATCGTTAG
AGATTTGACCGAAGATGGTATTGGTCATCCTGAAGTTGGTGATGCTGTTGCTAGATTGAA
AGAAGTCTTGCAATACAACGCTCCAGGTGGTAAATGTAATAGAGGTTTGACTGTTGTTG
CTGCCTACAGAGAATTGTCTGGTCCAGGTCAAAAAGATGCTGAATCTTTGAGATGTGCTT
TGGCTGTTGGTTGGTGCATTGAATTATTTCAAGCCTTCTTCTTGGTTGCCGATGACATTAT
GGATCAATCCTTGACTAGAAGAGGTCAATTGTGCTGGTACAAGAAAGAAGGTGTTGGTT
TGGACGCTATTTGGGACTCTTTTTTGTTGGAATCCTCCGTTTACAGAGTCTTGAAGAAGT
ACTGTAGACAAAGACCTTACTACGTCCACTTGTTAGAATTATTCTTGCAAACCGCTTACC
AAACCGAATTGGGTCAAATGTTGGATTTGATTACTGCCCCAGTTTCCAAGGTTGATTTGT
CTCATTTTTCCGAAGAAAGATACAAGGCCATCGTTAAGTACAAGACTGCTTTCTACTCTT
TTTACTTGCCAGTTGCTGCTGCTATGTACATGGTTGGTATTGACTCTAAAGAAGAACACG
AAAACGCTAAGGCTATCTTGTTGGAAATGGGTGAATACTTCCAAATCCAAGATGATTAC
TTGGATTGCTTTGGTGATCCAGCATTGACTGGTAAAGTTGGTACTGATATCCAAGACAAC
AAATGCTCTTGGTTGGTTGTTCAATGCTTGCAAAGAGTTACTCCAGAACAAAGACAATT
ATTGGAAGATAACTACGGTAGAAAAGAACCAGAAAAGGTTGCCAAGGTCAAAGAATTA
TATGAAGCCGTTGGTATGAGAGCTGCCTTTCAACAATATGAAGAATCCTCCTACAGAAG
ATTACAAGAATTGATCGAAAAGCACTCCAACAGATTGCCAAAAGAAATCTTTTTGGGTT
TGGCCCAAAAGATCTACAAGAGACAAAAGTGA

>*GPPS-SKL*

ATGGAATTCGACTTCAACAAGTACATGGATTCTAAGGCTATGACCGTTAACGAAGCTTT
GAACAAAGCTATCCCATTGAGATACCCACAAAAGATCTACGAATCCATGAGATATTCTT
TGTTGGCTGGTGGTAAAAGAGTTAGACCAGTTTTGTGTATTGCTGCCTGTGAATTGGTTG
GTGGTACTGAAGAATTGGCTATTCCAACTGCTTGTGCCATTGAAATGATTCACACTATGT
CCTTGATGCACGATGATTTGCCATGCATTGATAACGATGACTTGAGAAGAGGTAAGCCA
ACTAACCATAAGATCTTCGGTGAAGATACTGCTGTTACTGCTGGTAATGCTTTACATTCT

TACGCCTTCGAACATATTGCTGTCTCTACTTCTAAAACCGTTGGTGCCGATAGAATCTTG
AGAATGGTTTCTGAATTGGGTAGAGCTACTGGTTCTGAAGGTGTTATGGGTGGTCAAAT
GGTTGATATTGCTTCAGAAGGTGATCCATCCATTGACTTGCAAACTTTGGAATGGATTCA
TATCCATAAGACCGCCATGTTGTTGGAATGTTCTGTTGTTTGTGGTGCTATTATTGGTGGT
GCTTCCGAAATCGTTATTGAAAGAGCTAGAAGATACGCTAGATGCGTTGGTTTGTTGTTC
CAAGTTGTTGATGATATCTTGGACGTCACCAAGTCATCTGATGAATTAGGTAAAACCGCT
GGTAAGGATTTGATTTCTGATAAGGCTACTTACCCAAAGTTGATGGGTTTGGAAAAGGC
CAAAGAATTCTCCGACGAATTATTGAATAGAGCCAAGGGTGAATTGTCTTGTTTCGATCC
AGTTAAGGCTGCTCCATTATTGGGTTTGGCTGATTATGTTGCTTTCAGACAAAACTCTAA
GTTGTAA

>*GES-SKL*

ATGGAAGAATCCTCCAGTAAGAGAAGAGAATACTTGTTGGAAGAAACCACCAGAAAGT
TGCAAAGAAACGATACCGAATCCGTCGAAAAGTTGAAGTTGATCGATAACATCCAACAA
TTGGGTATCGGTTACTACTTCGAAGATGCTATTAACGCCGTTTTGAGATCTCCATTTTCTA
CCGGTGAAGAAGATTTGTTTACTGCTGCTTTGAGATTCAGATTATTGAGACACAACGGTA
TCGAAATCTCCCCAGAAATTTTCTTGAAGTTCAAGGACGAAAGAGGTAAGTTCGACGAA
TCTGATACTTTGGGTTTGTTGTCCTTGTACGAAGCTTCTAATTTGGGTGTTGCTGGTGAAG
AAATATTGGAAGAAGCTATGGAATTCGCTGAAGCCAGATTGAGAAGATCTTTGTCTGAA
CCAGCTGCTCCATTGCATGGTGAAGTTGCTCAAGCTTTGGATGTTCCAAGACATTTGAGA
ATGGCTAGATTGGAAGCCAGAAGATTCATTGAACAATACGGTAAGCAATCCGATCACGA
TGGTGATTTGTTGGAATTGGCTATCTTGGACTACAATCAAGTTCAAGCCCAACACCAATC
TGAATTGACCGAAATTATCAGATGGTGGAAAGAATTGGGTTTGGTCGATAAGTTGTCTTT
CGGTAGAGATAGACCATTGGAATGTTTCTTGTGGACTGTTGGTTTGTTGCCAGAACCTAA
ATACTCCTCCGTTAGAATTGAATTGGCCAAGGCCATTTCCATCTTGTTGGTTATTGATGA
TATCTTCGACACCTACGGTGAAATGGATGATTTGATTTTGTTCACCGATGCCATCAGAAG
ATGGGATTTGGAAGCAATGGAAGGTTTGCCAGAATATATGAAGATTTGCTACATGGCCT
TGTACAACACTACCAACGAAGTTTGTTACAAGGTTTTGAGAGACACTGGTAGAATCGTC
TTGTTGAATTTGAAGTCCACCTGGATCGATATGATCGAAGGTTTTATGGAAGAAGCCAA
GTGGTTTAATGGTGGTTCTGCTCCAAAGTTAGAAGAATATATCGAAAACGGTGTTTCTAC
CGCTGGTGCTTATATGGCTTTTGCCCACATATTTTTCTTGATCGGTGAAGGTGTTACCCAC
CAAAACTCTCAATTATTCACCCAAAAACCATACCCAAAGGTTTTTCTGCTGCCGGTAGA

ATTTTGAGATTGTGGGATGACTTGGGTACTGCCAAAGAAGAACAAGAAAGAGGTGATTT
GGCATCCTGTGTCCAATTATTCATGAAGGAAAAGTCCTTGACCGAAGAAGAAGCCAGAT
CTAGAATTTTGGAAGAAATCAAGGGTTTGTGGAGAGACTTGAATGGTGAATTGGTTTAC
AACAAGAACTTGCCATTGTCCATTATCAAGGTTGCTTTGAATATGGCTAGAGCCTCTCAA
GTTGTTTACAAGCACGATCAAGATACCTACTTCTCCTCTGTTGATAACTACGTTGATGCT
TTGTTCTTCACCCAATCCAAGTTGTGA

>P*TEF1*

CCCACACACCATAGCTTCAAAATGTTTCTACTCCTTTTTTACTCTTCCAGATTTTCTCGGA
CTCCGCGCATCGCCGTACCACTTCAAAACACCCAAGCACAGCATACTAAATTTCCCCTCT
TTCTTCCTCTAGGGTGTCGTTAATTACCCGTACTAAAGGTTTGGAAAAGAAAAAAGAGA
CCGCCTCGTTTCTTTTTCTTCGTCGAAAAAGGCAATAAAAATTTTTATCACGTTTCTTTTT
CTTGAAAATTTTTTTTTTTGATTTTTTTCTCTTTCGATGACCTCCCATTGATATTTAAGTTA
ATAAACGGTCTTCAATTTCTCAAGTTTCAGTTTCATTTTTCTTGTTCTATTACAACTTTTTT
TACTTCTTGCTCATTAGAAAGAAAGCATAGCAATCTAATCTAAGTTT

>T*TEF1*

TCAGTACTGACAATAAAAAGATTCTTGTTTTCAAGAACTTGTCATTTGTATAGTTTTTTTA
TATTGTAGTTGTTCTATTTTAATCAAATGTTAGCGTGATTTATATTTTTTTTCGCCTCGAC
ATCATCTGCCCAGATGCGAAGTTAAGTGCGCAGAAAGTAATATCATGCGTCAATCGTAT
GTGAATGCTGGTCGCTATACTG

>P*HIS3*

TAAATTCCCGTTTTAAGAGCTTGGTGAGCGCTAGGAGTCACTGCCAGGTATCGTTTGAAC
ACGGCATTAGTCAGGGAAGTCATAACACAGTCCTTTCCCGCAATTTTCTTTTTCTATTAC
TCTTGGCCTCCTCTAGTACACTCTATATTTTTTTATGCCTCGGTAATGATTTTCATTTTTTT
TTTTCCACCTAGCGGATGACTCTTTTTTTTTCTTAGCGATTGGCATTATCACATAATGAAT
TATACATTATATAAAGTAATGTGATTTCTTCGAAGAATATACTAAAAAATGAGCAGGCA
AGATAAACGAAGGCAAAG

>T*HIS3*

TGACACCGATTATTTAAAGCTGCAGCATACGATATATATACATGTGTATATATGTATACC
TATGAATGTCAGTAAGTATGTATACGAACAGTATGATACTGAAGATGACAAGGTAATGC
ATCATTCTATACGTGTCATTCTGAACGAGGCGCGCTTTCCTTTTTTCTTTTTGCTTTTTCTT
TTTTTTTCTCTTGAACTCGACGGATCATA

>P_{CYC1}

ATTTGGCGAGCGTTGGTTGGTGGATCAAGCCCACGCGTAGGCAATCCTCGAGCAGATCC
GCCAGGCGTGTATATATAGCGTGGATGGCCAGGCAACTTTAGTGCTGACACATACAGGC
ATATATATATGTGTGCGACGACACATGATCATATGGCATGCATGTGCTCTGTATGTATAT
AAAACTCTTGTTTTCTTCTTTTCTCTAAATATTCTTTCCTTATACATTAGGACCTTTGCAG
CATAAATTACTATACTTCTATAGACACGCAAACACAAATACACACACTAATC

>T_{CYC1}

ATGTAATTAGTTATGTCACGCTTACATTCACGCCCTCCCCCCACATCCGCTCTAACCGAA
AAGGAAGGAGTTAGACAACCTGAAGTCTAGGTCCCTATTTATTTTTTTATAGTTATGTTA
GTATTAAGAACGTTATTTATATTTCAAATTTTTCTTTTTTTTCTGTACAGACGCGTGTACG
CATGTAACATTATACTGAAAACCTTGCTTGAGAAGGTTTTGGGACGCTCGAAGGCTTTA
ATTTGC

References

Agrawal, G., and Subramani, S. (2013). Emerging role of the endoplasmic reticulum in peroxisome biogenesis. *Front. Physiol.* 4, 286. doi:10.3389/fphys.2013.00286.

Lin, C.-J., Sasse, C., Gerke, J., Valerius, O., Irmer, H., Frauendorf, H., et al. (2015). Transcription factor SomA is required for adhesion, development and virulence of the human pathogen *Aspergillus fumigatus*. *PLOS Pathog.* 11, e1005205. doi:10.1371/journal.ppat.1005205.

Motley, A. M., Nuttall, J. M., and Hettema, E. H. (2012). Atg36: the *Saccharomyces cerevisiae* receptor for pexophagy. *Autophagy* 8, 1680–1681. doi:10.4161/auto.21485.

Petroi, D., Popova, B., Taheri-Talesh, N., Irniger, S., Shahpasandzadeh, H., Zweckstetter, M., et al. (2012). Aggregate clearance of α-synuclein in *Saccharomyces cerevisiae* depends more on autophagosome and vacuole function than on the proteasome. *J. Biol. Chem.* 287, 27567–27579. doi:10.1074/jbc.M112.361865.

Schindelin, J., Arganda-Carreras, I., Frise, E., Kaynig, V., Longair, M., Pietzsch, T., et al. (2012). Fiji: An open-source platform for biological-image analysis. *Nat. Methods* 9, 676–682. doi:10.1038/nmeth.2019.

Shahpasandzadeh, H., Popova, B., Kleinknecht, A., Fraser, P. E., Outeiro, T. F., and Braus, G. H. (2014). Interplay between sumoylation and phosphorylation for protection against α-synuclein inclusions. *J. Biol. Chem.* 289, 31224–31240. doi:10.1074/jbc.M114.559237.

Wingler, L. M., and Cornish, V. W. (2011). Reiterative Recombination for the in vivo assembly of libraries of multigene pathways. *Proc. Natl. Acad. Sci. U. S. A.* 108, 15135–15140. doi:10.1073/pnas.1100507108.

Yan, M., Rayapuram, N., and Subramani, S. (2005). The control of peroxisome number and size during division and proliferation. *Curr. Opin. Cell Biol.* 17, 376–383. doi:10.1016/j.ceb.2005.06.003.
